# A Small Molecule Drug-Based Ru(II) Polypyridine Mass-Tag for Multimodal Imaging of Tissue Samples

**DOI:** 10.1021/acscentsci.5c01381

**Published:** 2025-10-21

**Authors:** Mihyun Park, Melina Rumpf, Guillermo Moreno-Alcántar, Manuel Seiler, Lieby Zborovsky, Katja Steiger, Susanne Kossatz, Angela Casini, Nicole Strittmatter

**Affiliations:** † Chair of Medicinal and Bioinorganic Chemistry, Department of Chemistry, School of Natural Sciences, 9184 Technical University of Munich, 85748 Garching, Germany; ‡ Professorship for Analytical Chemistry, Department of Biosciences, School of Natural Sciences, Technical University of Munich, 85748 Garching, Germany; § Catalysis Research Center Analytic Core Facility, Department of Chemistry, School of Natural Sciences, Technical University of Munich, 85748 Garching, Germany; ∥ Comparative Experimental Pathology, Institute of Pathology, School of Medicine and Health, Technical University of Munich, 81675 Munich, Germany; ⊥ Department of Nuclear Medicine, TUM University Hospital, Central Institute for Translational Cancer Research (TranslaTUM), School of Medicine and Health, Technical University of Munich, 81675 Munich, Germany

## Abstract

Mass spectrometry imaging (MSI) is a powerful tool for spatially resolved multiomics analysis of tissue samples in clinical research. However, its proteomics application is still limited due to challenges such as low ionization efficiency and signal interference from complex tissue environments. On-tissue mass-tag labeling (OTMT) addresses these limitations using affinity-based imaging agents that incorporate cleavable, highly ionizable reporter groups known as mass-tags (MTs). The majority of existing MTs rely on antibodies as targeting elements and organic moieties as reporter groups. Here, we introduce a new class of MTs featuring small-molecule inhibitors as binding motifs. Specifically, we present **PARPi-MT**, composed of a photocleavable and luminescent Ru­(II)-based reporter and the poly­(ADP-ribose) polymerase (PARP) inhibitor Olaparib for the targeted bimodal imaging of PARP1 in H446 xenograft tumor and mouse brain sections, via desorption electrospray ionization (DESI)-MSI and fluorescence microscopy. Using small-molecule inhibitors as binding motifs expands the design versatility and potential applications of OTMT, while overcoming some of the challenges of antibody-based mass-tags. The Ru­(II)-based reporter group offers further advantages, including distinct isotopic signatures derived from the metal center and inherent multimodal imaging capabilities.

## Graphical abstract

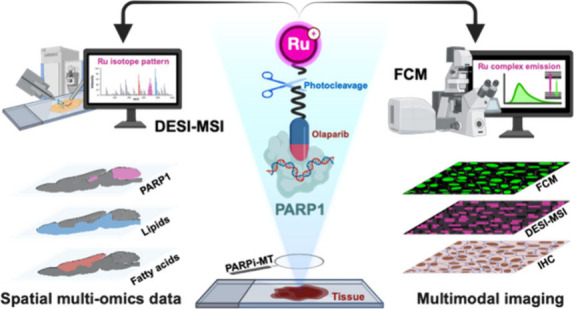

## Introduction

Understanding the spatial architecture of tissues and how biomolecules interact within their native microenvironment can provide critical insights into cellular function, disease progression, and therapeutic response. Traditional bulk omics approaches, while powerful, inherently lose information on cellular context and heterogeneity by averaging signals across heterogeneous cell populations. In contrast, Mass Spectrometry Imaging (MSI) preserves the spatial context while enabling comprehensive, multiomics analysis of tissue samples.^1–3^ MSI is an *ex vivo* surface imaging technique and can visualize the spatial distribution of diverse endogenous molecules within tissue sections in a single measurement (Figure 1). Depending on the ionization source and the instrumental setup, minimal to no sample preparation is required.^4^ Additionally, samples can be measured at varying resolutions without sample destruction, allowing the same sample to be used for further histological assessments, such as immunohistochemistry (IHC) or immunofluorescence (IF), which is crucial for precious clinical samples.

**Figure 1:**
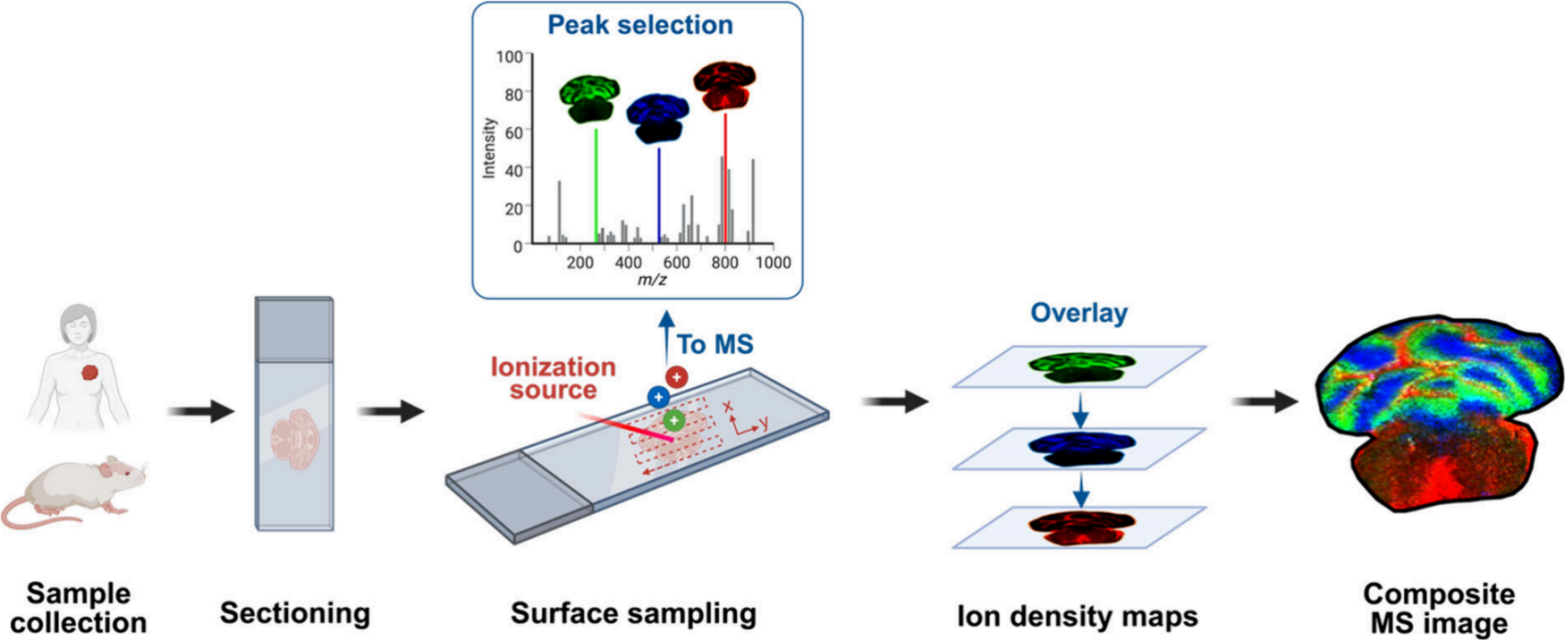
Schematic overview of the general workflow for MSI. Created with BioRender.com/ukja1gi.

While MSI has seen widespread application in metabolomics and lipidomics, mainly due to the high ionization efficiency of small molecules, its extension to direct analysis of proteins and other high-molecular-weight molecular classes remains limited. This is primarily due to the physicochemical complexity of larger biomolecules, resulting in low ionization efficiency, poor desorption of intact proteins, ion suppression by abundant small molecules, and insufficient sensitivity for low-abundance targets. As a result, spatial proteomics using MSI has proven challenging to implement effectively. To address these limitations, several strategies have been developed over the past two decades, with on-tissue enzymatic digestion (OTED)^5^ and on-tissue mass-tag labeling (OTMT)^6^ being the most prominent. In OTED, tissue sections are enzymatically treated to break down proteins into peptide fragments that are detectable by MSI.^5^ However, assigning these peptide mass fingerprints (PMFs) to parent proteins is complex due to nontargeted enzymatic digestion and difficulty distinguishing endogenous peptides from PMFs. In contrast, OTMT is a targeted method that uses affinity-based imaging agents known as mass-tags (MTs) to visualize biomolecules within tissue sections (Figure 2).^6–8^ MTs are typically composed of a target-specific binding motif linked to a cleavable and highly ionizable reporter group to facilitate detection by MS. Photocleavable MTs are predominantly used,^9–11^ as they are compatible with laser-based ionization methods such as matrix-assisted laser desorption ionization (MALDI), which are widely employed in clinical research settings. Following incubation of the tissue section with a MT solution and subsequent washing to remove unbound tags, the reporter group is released upon irradiation and detected at the site of target binding. Unlike IHC and IF, which have limited multiplexing capacity and multimodal applicability, OTMT offers the potential for near-unlimited multiplexing, as any cleavable and ionizable molecule can be utilized as a reporter group as long as it results in a distinct *m*/*z* signal. Additionally, OTMT can retain the multiomics and multimodal capability of MSI using refined washing and sample handling protocols.^12,13^

**Figure 2:**
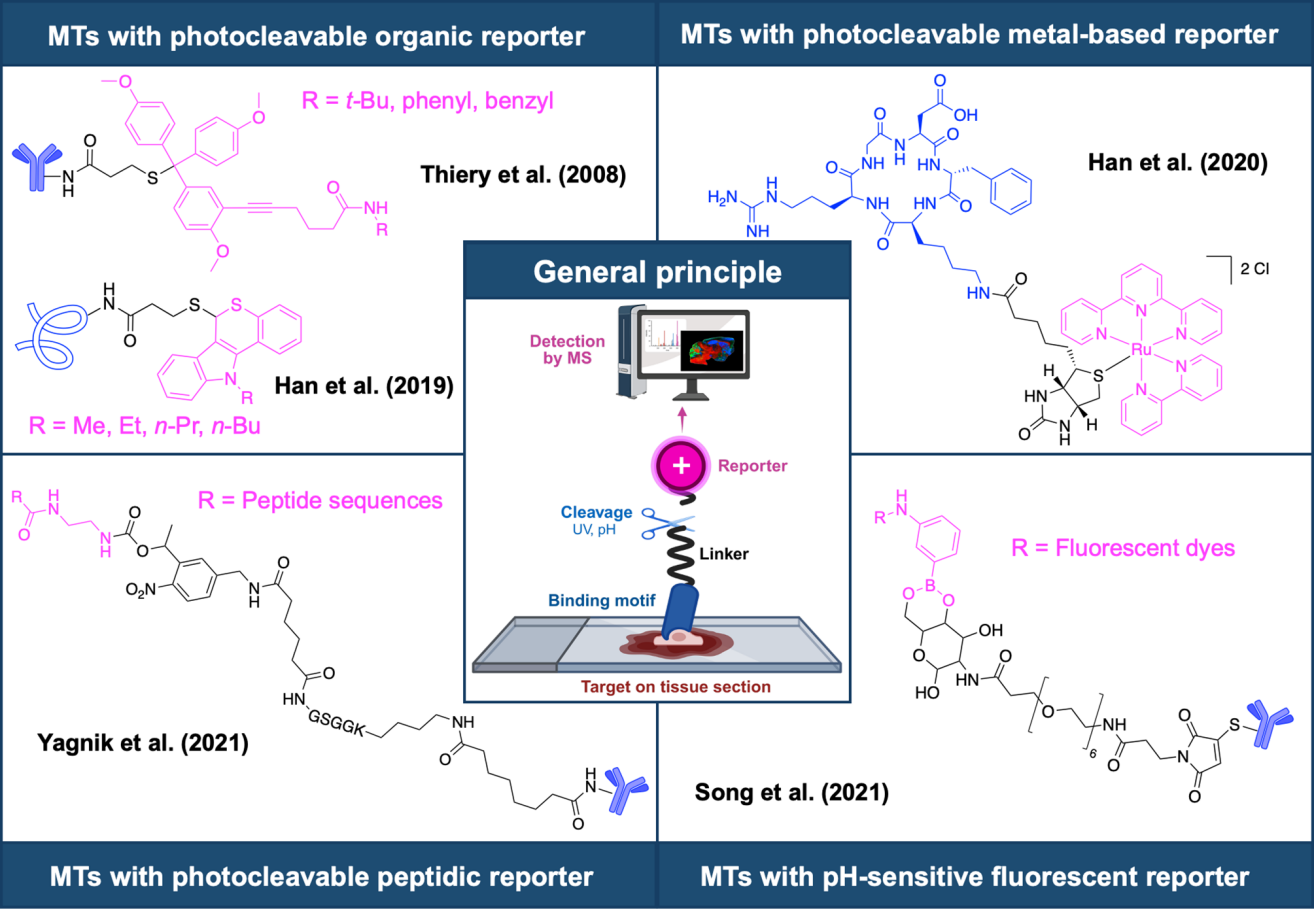
Schematic representation of the OTMT concept,^6^ and selection of photocleavable^9,11,14,22^ and chemocleavable MTs.^16^ Figure depicting the general principle was created with BioRender.com/n0dmjoi.

Depending on the type of reporter group, MTs can be classified into different categories, with organic molecules representing the most widely used class to date (Figure 2),^6,9,11,14^ although metal-conjugated probes in conjunction with elemental MSI are also well established.^15^ Thiery et al. first introduced MTs employing secondary antibodies (Abs) functionalized with photocleavable trityl groups for MALDI-MSI applications.^9^ Over the years, the concept of MTs for MSI applications has advanced further, recently leading to the development of commercially available MTs by AmberGen Inc. (MA, USA) for MALDI-MSI applications, consisting of Abs modified with photocleavable peptidic reporter groups.^14^ Song et al. recently extended the MT concept to nonlaser-based ionization methods such as desorption electrospray ionization (DESI) through pH-sensitive cleavable linkers (Figure 2).^16^

In terms of binding motif, Abs remain the predominant choice for target-specific recognition,^6,9,14,16^ leveraging well-established methodologies derived from IHC. While Abs offer high specificity and affinity, their use is associated with several drawbacks, including high production costs, complex synthesis and purification processes, and stringent handling requirements.^17^ Peptides have emerged as an alternative approach due to their smaller size and ease of customization.^11^ However, their synthesis can still be costly and technically demanding, especially when tailored for specific protein targets. Here, we propose to use small-molecule drugs, particularly established pharmacological inhibitors, as selective binding motifs. Unlike Abs, the production and handling of such small molecules are less complicated, while specificity is given by their function as selective inhibitors. Beyond commercial inhibitors, this approach can draw on the vast pharmaceutical compound libraries, including millions of well-characterized compounds that never reached the market, providing a rich source of potential binders with known bioactivity. Small molecule binding motifs have been successfully applied in radiotracer development,^18–20^ yet the concept remains untapped in the context of MTs. Thus, establishing such drugs as a new binding motif class could significantly expand the toolkit of targeted MSI, enabling more robust and versatile approaches for spatial proteomics.^21^

Here, we designed the first MT that uses a small molecule drug, specifically Olaparib (Lynparza), as the binding moiety for MSI of proteins. Olaparib inhibits selectively both poly­(ADP-ribose) polymerase 1 and 2 (PARP1/2).^23^ However, PARP1 is believed to account for more than 90% of the total cellular PARP activity and is the most abundantly expressed PARP across many tissues and can thus be considered as the main contributor to observed protein localizations.^24,25^ PARP enzymes are responsible for sensing damaged DNA and catalyzing its repair and are overexpressed in various cancer types. High expression is further associated with tumor deterioration, metastasis and angiogenesis.^24^ Olaparib has previously been proven to be a versatile PARP-targeting moiety for applications including radiotherapy, nuclear and fluorescence imaging (FI), as well as in the design of proteolysis targeting chimaeras (PROTACs).^23,26,27^

As the reporter unit, we selected a photocleavable Ru­(II) polypyridyl complex [Ru­(phen)­(tpy)]^2+^ (phen = 1,10-phenanthroline, tpy = 2,2′:6′,2″-terpyridine), similar to the one that we have previously integrated in an α_ν_β_3_ integrin targeting MT, deploying a peptide binding motif (Figure 2).^11^ Upon photoexcitation, ruthenium polypyridyl compounds of this family are known to selectively photosubstitute one ligand of the coordination sphere by a solvent molecule.^28–30^ This photoreactivity is not present (or extremely rare) in other metal complexes. As for the organic MTs, the ‘uncaging’ of the Ru­(II) MT upon UV-light activation is pivotal to facilitate its detection, providing a *fingerprint signal* in the MS spectrum.^11^ Unlike organic reporter groups, which can be challenging to differentiate from endogenous molecules, ruthenium offers a unique isotopic pattern distribution that facilitates unambiguous identification in complex MSI data sets. Moreover, the Ru­(II) moiety is positively charged, easily ionizable and amenable to ligand modification, enabling potential multiplexing capabilities. In addition, Ru­(II) polypyridyl complexes are well-known for their emissive properties and have been applied as fluorescence imaging tags in various biological settings.^31^ The applicability of our Ru­(II) MT for targeted bimodal imaging of PARP1 was assessed in H446 xenograft tumor and mouse brain sections, via DESI-MSI and confocal fluorescence microscopy.

## Results and Discussion

### PARPi-MT Design, Synthesis, and Characterization

**PARPi-MT** features a polypyridyl Ru­(II) moiety, specifically [Ru­(phen)­(tpy)]^2+^, as the reporter. This moiety is linked via photocleavable metal–ligand coordination to a D-biotin linker tethered to the Olaparib binding motif (Scheme 1). Thioether ligands bound to Ru­(II) polypyridyl complexes are efficiently cleaved from the metal center by visible light irradiation.^32^

**Scheme 1:**
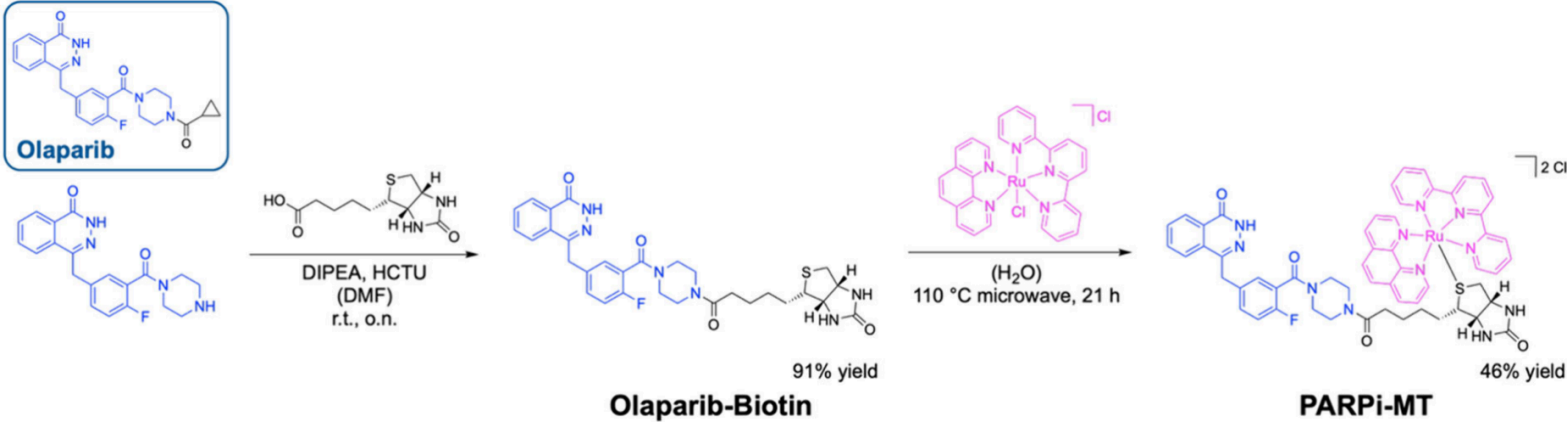
Synthesis Pathway of **PARPi-MT** ^a^

a In the first step, an Olaparib-derived binding motif was conjugated to D-biotin through an amidation reaction, resulting in the Olaparib-Biotin conjugate. In the following step, this conjugate was reacted with [RuCl­(phen)­(tpy)]Cl via a ligand exchange reaction, achieving **PARPi-MT**. PARP1/2 binding motif is highlighted in blue, linker in black, and photocleavable reporter group in pink. The structure of Olaparib is depicted for comparison.

**PARPi-MT** was synthesized via a two-step procedure (see for details). In the first step, the Olaparib-derived binding motif was conjugated to D-biotin through an amidation reaction, yielding the Olaparib-Biotin conjugate in 91% yield. Afterward, the conjugate was reacted with [RuCl­(phen)­(tpy)]­Cl via a ligand exchange reaction, in which the chloride ligand was displaced by the thioether group of D-biotin. The reaction was carried out in an aqueous solution at 110 °C, affording **PARPi-MT** in 46% isolated yield.

The compounds were characterized by NMR spectroscopy (Figures S1–S14), heated electrospray ionization (HESI) mass spectrometry (Figures S17–S20), and absorption and emission spectroscopy (Figures S21). The UV–vis absorption spectrum of **PARPi-MT** in THF presented a metal-to-ligand charge transfer (MLCT) band at λ_max_ = 459 nm, which is blue-shifted compared to [RuCl­(phen)­(tpy)]Cl (λ_max_ = 504) (Figure S21A), in line with previously reported thioether-bound Ru­(II) complexes.^32^ In the emission spectra, [RuCl­(phen)­(tpy)]Cl and **PARPi-MT** exhibited similar broad unstructured emissions with maxima at 617 and 620 nm, respectively (Figure S21B).

To assess the stability of **PARPi-MT** in aqueous conditions, ^1^H NMR spectra of the compound (3.5 μM) were recorded at room temperature (r.t.) in D_2_O at defined time intervals over 24 h (Figure S15). In the absence of light, no noticeable decay was observed, indicating that **PARPi-MT** remains stable under these conditions. This result aligns with previously reported thioether-bound Ru complexes.^32^ Furthermore, a complementary stability study conducted under aqueous conditions in the presence of an excess amount of *N*-acetyl cysteine (NAC), used as a model nucleophile, confirmed the high stability of **PARPi-MT** (Figure S16), showing no change over a 24 h period.

The photocleavage efficiency of **PARPi-MT** was also qualitatively assessed by irradiating a solution of the MT at 420 nm and monitoring the spectral changes over time (Figure S22). UV–vis absorption spectra were recorded at defined total irradiation times (*t* = 0, 10, 20, 30, 60, 90, 120, 180, and 240 s) to track the progression of photocleavage. Photoconversion was indicated by a red shift in the absorption maximum, consistent with the formation of the aqua complex [Ru­(H_2_O)­(phen)­(tpy)]^2+^, as previously reported in the literature.^32^ To validate this assignment, a solution of [RuCl­(phen)­(tpy)]­Cl was measured before and after 240 s of irradiation under identical conditions. Complete conversion of **PARPi-MT** to the aqua complex was observed after 60 s, as evidenced by the appearance of an absorption maximum at 474 nm, matching that of the irradiated [RuCl­(phen)­(tpy)]­Cl solution.

In addition, the PARP1 inhibitory properties of **PARPi-MT** were determined using a chemiluminescent PARP1 assay (Figures S23), which allowed the calculation of the half-maximal inhibitory concentration (IC_50_) of **PARPi-MT** and its pegylated derivative (**PARPi-PEG3-MT**) in comparison to the benchmark compounds, Olaparib and AZD5305 (Saruparib). The obtained results showed that **PARPi-MT** is a potent PARP1 inhibitor, even outperforming Olaparib and AZD5305 by 2.3- and 1.5-fold, respectively, while **PARPi-PEG3-MT** showed a comparable IC_50_ to Olaparib (Table 1). Since the pegylated derivative was shown to have lower affinity for PARP1 compared to **PARPi-MT**, we decided to perform MSI only with the latter compound.

**Table 1:**
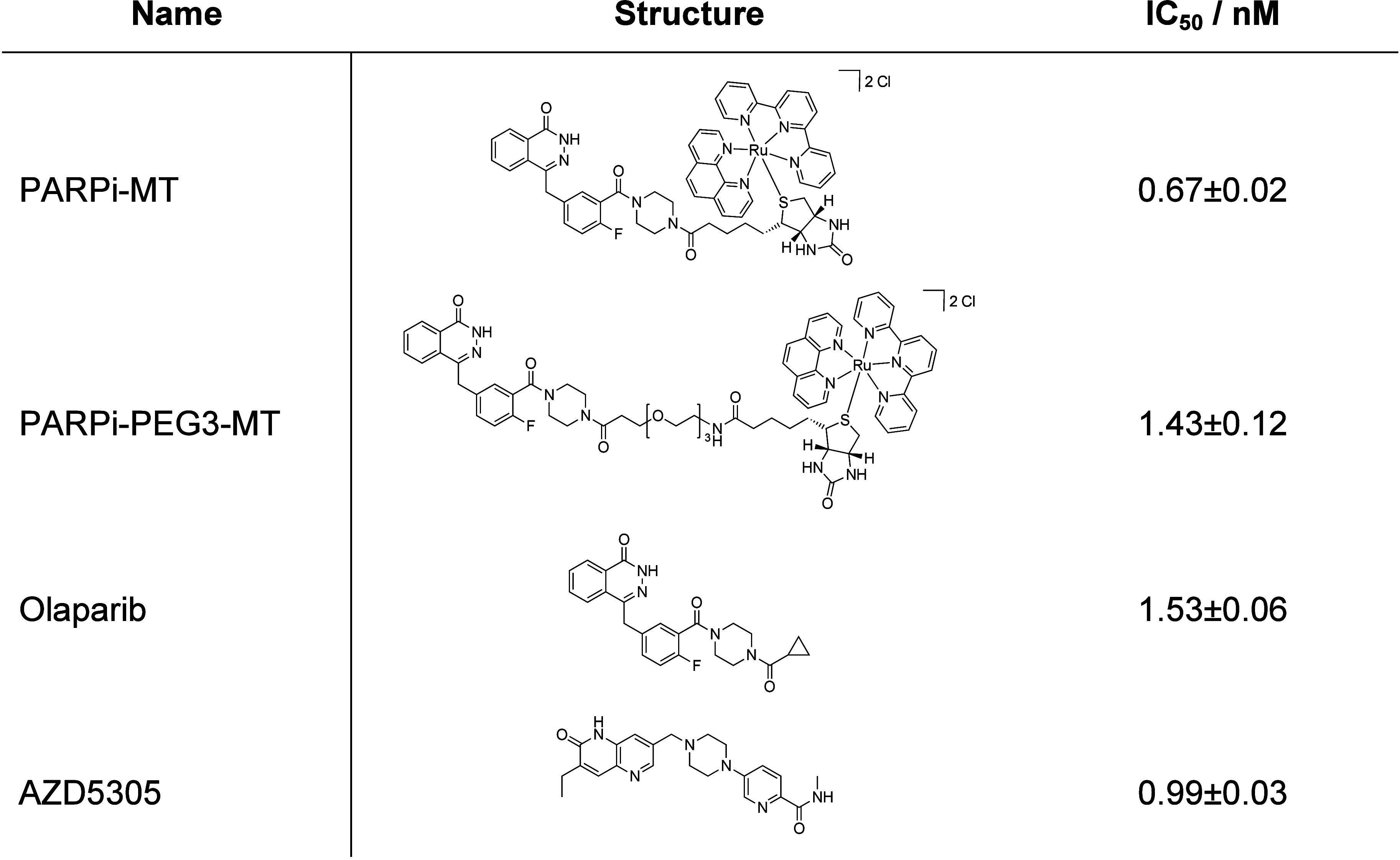
PARP1 Inhibitory Activity (IC_50_) of **PARPi-MT** and **PARPi-PEG3-MT** in Comparison to the Benchmarks, Olaparib, and AZD5305 (*n* = 2)

### PARP1 Imaging in Tissue Sections Using PARPi-MT

Subsequently, **PARPi-MT** was evaluated by DESI-MSI for its ability to visualize PARP1 in fresh-frozen tissue sections. As a nuclear enzyme, PARP1 is ubiquitously expressed across tissues. However, to demonstrate the specificity of **PARPi-MT**, two model tissues were selected: Mouse brain and a xenograft tumor derived from the human small cell lung carcinoma (SCLC) cell line NCI-H446. These tissues exhibit distinct heterogeneous levels and spatial distributions of PARP1. While the SCLC tumor tissue was selected based on its high expression levels of PARP1,^33^ the mouse brain was selected due to its different PARP1 expression patterns over the different brain areas.^34,35^ The tissue sections were stained with **PARPi-MT** solution (0.2 mg/mL in phosphate-buffered saline (PBS) buffer, pH 7.4) for 45 min at r.t. and then washed in NAC-containing PBS (0.07 mg/mL), PBS, and 1% trifluoroacetic acid (TFA) solution (Figure 3). TFA was added to the last wash step to force the reporter group into the TFA complex, decreasing spectral complexity and increasing sensitivity. Once dried, the stained tissue sections were irradiated at 420 nm for 30 min to photocleave the Ru­(II) complex at the binding site prior to MS analysis.

**Figure 3:**
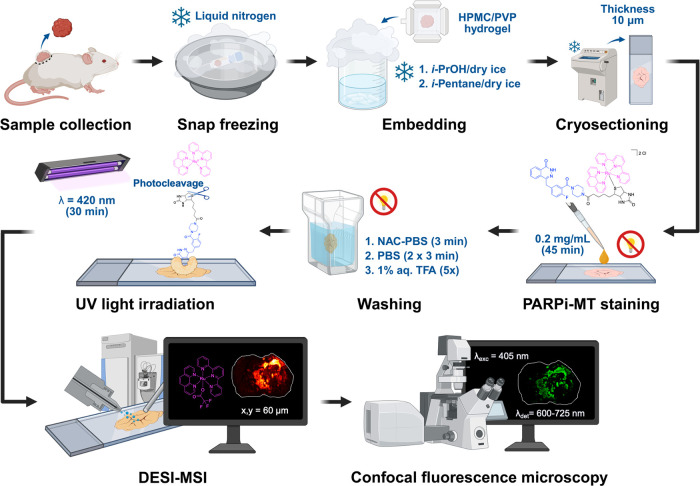
Comprehensive overview of the **PARPi-MT** staining and imaging workflow. Tissue samples are collected and snap-frozen in liquid nitrogen. The fresh frozen samples are embedded in a hydrogel composed of (hydroxypropyl)­methylcellulose (HPMC) and polyvinylpyrrolidone (PVP) and snap-frozen in *iso*-propanol (*i*-PrOH)/dry ice and *iso*-pentane/dry ice bath. The embedded samples are cryosectioned and stained in the dark with a **PARPi-MT** PBS solution. After washing off excess unbound **PARPi-MT** in NAC-PBS, PBS and 1% aq. TFA solution, the Ru­(II) reporter is photocleaved under UV light irradiation. The spatial distribution of the cleaved Ru­(II) reporter, indirectly reflecting PARP1 localization, is then visualized by DESI-MSI and confocal fluorescence microscopy. Figure was partially created with BioRender.com/q9qzgya.

The cleaved Ru­(II) reporter was detected as [Ru­(CF_3_COO)­(phen)­(tpy)]^+^ species by DESI-MSI. The spatial distribution of the adduct was compared to the corresponding IHC results for PARP1, the most abundant PARP enzyme, of adjacent tissue sections, showing a clear correlation (Figure 4). In the brain, increased expression was observed in the cerebellum, hippocampus, cerebral cortex, and olfactory bulb, which was consistent with data from the Human Protein Atlas.^34,35^ In the tumor section, overexpression was particularly evident at the interface to necrotic areas. This observation aligns with the literature,^24^ as PARP1 activates the apoptosis pathway in cancer cells, and overactive PARP1 leads to nicotinamide adenine dinucleotide (NAD^+^)/adenosine triphosphate (ATP) depletion-induced necrosis.

**Figure 4:**
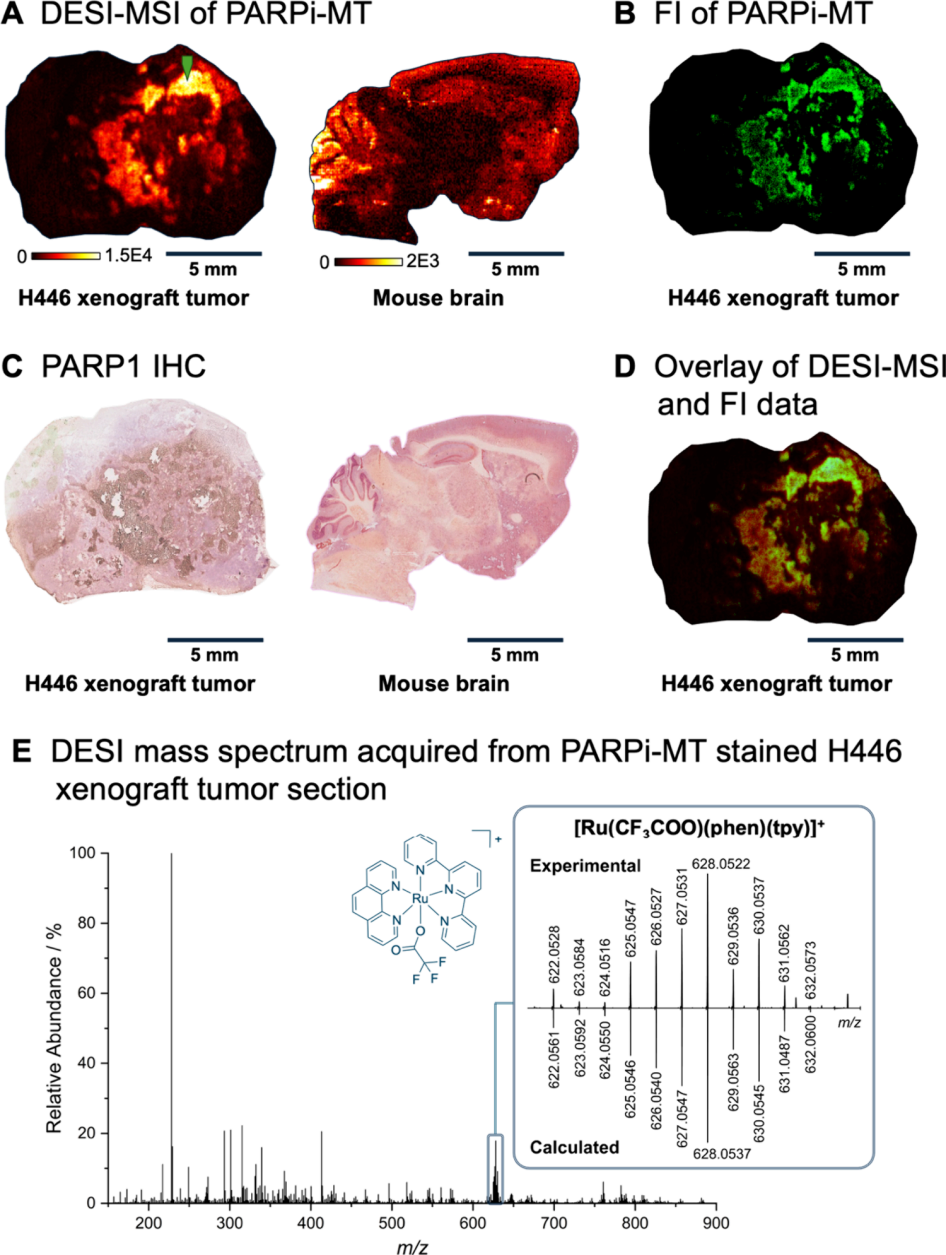
(A) DESI-MSI of a **PARPi-MT** stained H446 xenograft tumor and mouse brain section, depicting the spatial distribution of [Ru­(CF_3_COO)­(phen)­(tpy)]^+^ (*m*/*z* 628.0521 ± 3 ppm; total ion current (TIC)-normalized; spatial resolution: X,Y = 60 μm). (B) Confocal fluorescence microscopy image of the same H446 xenograft tumor section used for DESI-MSI, employing a 600–725 nm channel mode with false-color representation (green). (C) IHC of corresponding adjacent tissue sections, stained for PARP1. (D) Overlay of DESI-MSI (in red) and FI images (in green). (E) A DESI mass spectrum recorded at the marked spot (green arrow) of the H446 xenograft tumor shows the isotope pattern distribution of [Ru­(CF_3_COO)­(phen)­(tpy)]^+^ in comparison to the calculated isotopic pattern.

Given the heterogeneity and the variable expression levels of PARP1 across the analyzed tissue sections, a control experiment was performed to evaluate potential ion suppression effects of [Ru­(CF_3_COO)­(phen)­(tpy)]^+^ in different tissue regions and to confirm the specificity of **PARPi-MT**. For this purpose, a neighboring tissue section was uniformly spray-coated with a constant layer of an aqueous solution containing 10 μM [RuCl­(phen)­(tpy)]Cl and 10 μM TFA using a HTX TM-Sprayer. This coated section was then analyzed by DESI-MSI under identical conditions to those used for the **PARPi-MT** stained tissue section (). No region-specific ion suppression was observed, confirming that the observed variations in signal intensities of [Ru­(CF_3_COO)­(phen)­(tpy)]^+^ reflect the differences in PARP1 expression.

To test the suitability of the Ru­(II) reporter group as a bimodal imaging tool for both MSI and fluorescence imaging, the same tissue section from the MSI experiment was measured using confocal fluorescence microscopy. The resulting image showed a distribution comparable to that of DESI-MSI, confirming the bimodal applicability of Ru­(II) polypyridyl complexes as reporter groups (Figure 4). Since the spatial resolution in DESI-MSI is generally lower (typical resolution: 25–100 μm)^36–38^ than that of optical microscopy techniques, confocal microscopy could provide additional subcellular spatial information.^39^

### Retention of Endogenous Metabolites Post PARPi-MT Staining

Unlike IHC and IF, MSI has the potential for multiomics image acquisition.^40^ However, it is well-known that washing tissue sections can remove certain molecular classes, depending on the solvent composition and washing protocol employed.^41–44^ Thus, both **PARPi-MT** stained and native brain tissue sections were analyzed by MSI within a *m*/*z* range of 80–900 Da in positive ion mode to identify affected compound classes. Peaks were tentatively annotated based on exact mass through comparison with publicly available databases ().^45–47^ 397 metabolites were tentatively annotated and subsequently classified into *i)* peaks with increased intensity poststaining (197 *m*/*z* values) and *ii)* peaks with decreased intensity (200 *m*/*z* values). The following trends were observed: monoisotopic *m*/*z* values with increased intensities poststaining were predominantly observed in the 500–800 Da range, whereas no significant change was detected in the 200–400 Da range, and decreased intensities were primarily noted in the ranges of 80–200, 400–500, and 800–900 Da (Figure 5). When this trend was evaluated alongside potential database matches, it was concluded that smaller and more polar molecules, particularly amino acids, nucleobases, organic acids, metabolites, and their derivatives, tended to be washed out during the staining process. This is expected as the washing solutions are of an aqueous nature. In contrast, the detection of larger and more hydrophobic species, such as fatty acids, appeared to be similar or even slightly enhanced. These findings demonstrate that **PARPi-MT** stained tissue sections not only enable visualization of the target proteins but simultaneously enable imaging of a broad range of disease- and region-relevant biomolecules in a single measurement. This represents a significant advantage over traditional targeted methods like IHC and IF, which provide spatial information limited to the labeled target while losing broader molecular context. On the other hand, the commercially available MALDI-IHC workflow requires a sequential, two-step MALDI analysis to extract multiomics information from the same tissue section. The native tissue section is first analyzed for lipids via MALDI-MSI, followed by matrix removal, fixation, MT staining, matrix reapplication and a second MALDI analysis to visualize the stained targets.^14,48^ In contrast, our approach demonstrates that lipidomic and metabolic information can be partially retained after MT application, enabling simultaneous acquisition of untargeted and targeted spatial data from a single MSI run.

**Figure 5:**
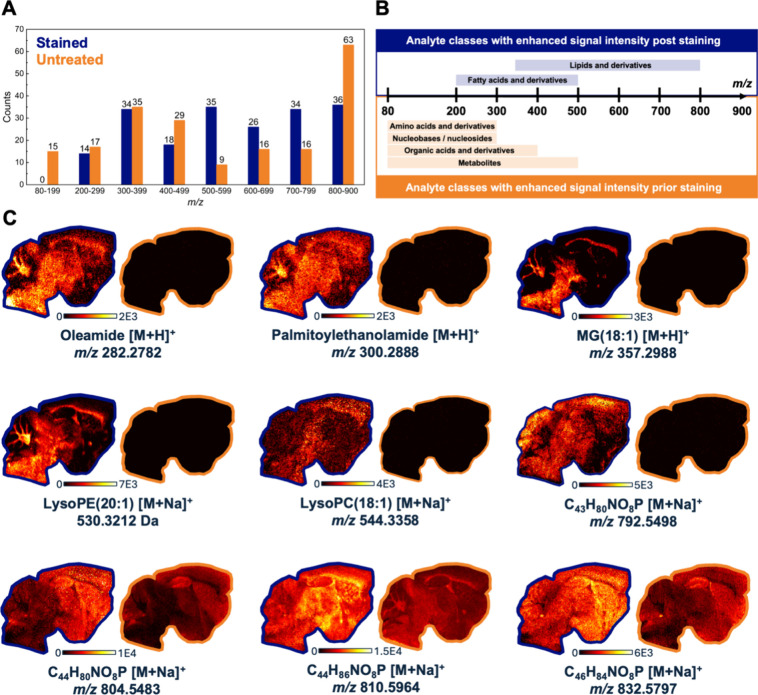
Comparative DESI-MSI analysis of **PARPi-MT** stained (blue) vs untreated (orange) mouse brain section. (A) Distribution of monoisotopic *m*/*z* peaks with elevated intensities in stained or untreated tissue sections across distinct *m*/*z* ranges. (B) Analyte classes significantly enriched in either stained or untreated sections. (C) Representative TIC-normalized ion density maps of identified endogenous molecules, acquired at *m*/*z* 80–900 within a ± 3 ppm *m*/*z* window and a spatial resolution of *X*,*Y* = 60 μm. Images of the stained section are framed in blue, while those of the untreated section are framed in orange.

## Conclusion

In this study, we introduced a novel class of bimodal metal-based MTs derived from a small-molecule drug inhibitor. We demonstrated that **PARPi-MT**, a Ru­(II)-based MT incorporating the PARP1 inhibitor Olaparib as the binding motif, enables the spatial visualization of PARP1 in murine brain and xenograft tumor tissues using both DESI-MSI and FI. These distributions matched those of a standard IHC analysis of a neighboring tissue section. The applied staining and washing protocols were shown to preserve hydrophobic endogenous molecular species, particularly fatty acids and lipids, making it a suitable staining agent for combined visualization of protein targets and the lipidome.

By employing small-molecule drugs as binding motifs and leveraging the structural flexibility of the Ru­(II) reporter moiety, this approach offers an alternative to Abs- and peptide-based systems, with strong potential for high multiplexing capabilities. In comparison to Abs, small molecules provide practical advantages, including simplified synthesis, improved storage stability, and reduced production costs, eliminating the need for specialized handling conditions, while preserving the necessary specificity. Further work will be devoted to the optimization of the Ru­(II) MTs for photosubstitution and multiplexing; therefore, we will focus on the family of [RuL­(N∧N)­(tpy)]^2+^ complexes, where N∧N is a variety of bidentate ligands, and L is a “caged” monodentate pyridyl ligand that can be released upon irradiation.^29^ These compounds have improved photosubstitution quantum yield, also benefiting from the introduction of steric hindrance around the metal center due to substitution on the N∧N ligand. In parallel, we will evaluate the effects of ligand substitution on the compounds’ photoluminescence quantum yield, to preserve FI capabilities.

Our findings also reinforce the suitability of Olaparib and other small-molecule drug inhibitors as targeting moieties and highlight the potential of small-molecule-directed, metal-based MTs for multimodal and multiplexed protein imaging using mass spectrometry. In the future, different disease-relevant enzymes could be targeted by our Ru­(II) MTs, exploiting the existing knowledge on selective inhibitors. For example, ongoing studies in our group are devoted to the design of Ru­(II) MTs incorporating small-molecule inhibitors of the fibroblast activation protein (FAP). FAP is a serine protease overexpressed on the cancer-associated fibroblasts of over 90% of epithelial tumors, such as breast, colorectal, head and neck, lung, ovarian, and pancreatic adenocarcinomas.^49^ Recently, FAP inhibitors (FAPi) have been successfully integrated in the design of radiopharmaceuticals for targeted theranostic applications,^50^ and a similar approach could be envisaged for the obtainment of oncotargeted Ru­(II) MTs. Overall, our study paves the way for the development of a broader MT toolkit, ultimately enhancing the diagnostic and analytical power of MSI in spatial proteomics.

## Supplementary Material





## Notes

NMR and HESI-MS spectra of Ru­(II) MTs, ^1^H NMR stability studies, UV–vis absorption spectra of [RuCl­(phen)­(tpy)]­Cl and **PARPi-MT**, qualitative assessment of the photocleavage efficiency of **PARPi-MT** by UV–vis absorption spectroscopy, PARP1 activity determinations (IC_50_), analysis of ion suppression of [Ru­(CF_3_COO)­(phen)­(tpy)]^+^ in distinct tissue regions of mouse brain and H69 xenograft tumor via DESI-MSI, and list of metabolites increased in mouse brain tissue section post **PARPi-MT** staining (Table S1) and additional experimental details, materials, and methods (PDF)
Transparent Peer Review report available (PDF)
