# Supplementary material for: A Small Molecule Drug-Based Ru(II) Polypyridine Mass-Tag for Multimodal Imaging of Tissue Samples
Source: ACS Cent Sci. 2025 Oct 21;11(11):2230–9. doi: 10.1021/acscentsci.5c01381 (PMC12670285; doi:10.1021/acscentsci.5c01381)
Supplement: Supplementary file 1 [file oc5c01381_si_001.pdf]

Supporting Information

A small molecule drug-based Ru(II) polypyridine mass-tag for multimodal imaging of tissue samples

Mihyun Park,[1,2] Melina Rumpf,[1,2] Guillermo Moreno-Alcántar,[1] Manuel Seiler,[3] Lieby Zborovsky,[2] Katja Steiger,[4] Susanne Kossatz,[5] Angela Casini,[1,*] Nicole Strittmatter[2,*]

[1] Chair of Medicinal and Bioinorganic Chemistry, Department of Chemistry, School of Natural Sciences, Technical University of Munich, 85748 Garching, Germany

[2] Professorship for Analytical Chemistry, Department of Biosciences, School of Natural Sciences, Technical University of Munich, 85748 Garching, Germany

[3] Catalysis Research Center Analytic Core Facility, Department of Chemistry, School of Natural Sciences, Technical University of Munich, 85748 Garching, Germany

[4] Comparative Experimental Pathology, Institute of Pathology, School of Medicine and Health, Technical University of Munich, 81675 Munich, Germany

[5] Department of Nuclear Medicine, TUM University Hospital, Central Institute for Translational Cancer Research (TranslaTUM), School of Medicine and Health, Technical University of Munich, 81675 Munich, Germany

* Corresponding authors: angela.casini@tum.de; nicole.strittmatter@tum.de

Materials and Methods

Microwave Reactions and Chromatography

Microwave reactions were conducted in Biotage® Initiator^{+} (Biotage AB, Uppsala, Sweden) and flash chromatography was conducted with Biotage® Isolera™ One (Biotage AB, Uppsala, Sweden). Semi-preparative RP-HPLC was carried out on Thermo Fisher Scientific UltiMate 3000 HPLC (Thermo Fischer, Scientific, Darmstadt, Germany) equipped with a semi-preparative MultoKrom 100–5 C18 column (250 x 20.0 mm, 5 µm particle size; CS-Chromatographie Service GmbH, Langerwehe, Germany) with a flow rate of 10 mL/min.

Nuclear Magnetic Resonance Spectroscopy

NMR spectra were recorded either on Bruker Ultrashield™ 400, Bruker Ascend™ 400, Bruker Avance Neo 400 or Bruker Avance III HD 500 (Bruker Corporation, Billerica, USA). Chemical shifts (δ) are given in parts per million and referenced to residual resonances of the respective deuterated solvent. MestReNova v14.2.1-27684 (Mestrelab Research S.L., Santiago de Compostela, Spain) was used for data processing. The observed signals were abbreviated as follows: s – singlet, d – doublet, dd – doublet of doublets, dt – doublet of triplets, t – triplet, q – quartet and m – multiplet.

High-Resolution Mass Spectrometry

HRMS were recorded on a Q Exactive™ Plus Hybrid Quadrupole-Orbitrap™ mass spectrometer (Thermo Fisher Scientific, Bremen, Germany) using a heated electrospray ionization (HESI) source and following parameters: 70,000 resolution at *m/z* 200, positive ion mode, 1 microscan, no lock mass, 1E6 AGC target, 50 ms maximum injection time, 6 sheath gas flow (N_2, N5.0 purity), 4 Aux gas flow (N_2, N5.0 purity), 3.8 kV spray voltage, 300 °C capillary temperature, 30 S-lens RF level, 100 °C Aux temperature, 20 µL/min spray flow rate. Thermo Xcalibur 3.0.63 (Thermo Fischer Scientific, Waltham, USA) and OriginPro 2021 9.8.0.200 (Origin Lab Corp., Northampton, USA) were used for data processing. Theoretical isotope fine structures were calculated with enviPat Web,[1] and created with OriginPro 2021 9.8.0.200.

UV-Vis Absorption and Emission Spectroscopy

Each sample was dissolved in H_2O (1 M) and diluted in THF to a final concentration of 5 µM. UV-Vis absorption measurements were acquired in a Cary 60 UV-Vis spectrophotometer (Agilent Technologies, Inc., Santa Clara, USA). For emission measurements, the solutions were saturated with argon. Emission spectra were recorded in a Cary Eclipse fluorescence spectrometer (Agilent Technologies, Inc., Santa Clara, USA). OriginPro 2021 9.8.0.200 (Origin Lab Corp., Northampton, USA) was used for data processing.

PARP1 IC_{50} Assay

Assays were performed by BPS Bioscience, San Diego, CA. PARPi-MT and PARPi-PEG3-MT were provided to BPS. Benchmark controls (Olaparib, AZD5305) were provided by BPS. Each measurement was performed in duplicates and in a test range of 0.000051 – 1 µM according to the BPS assay kit protocols (BPS Bioscience, #80551). Luminescence was measured using a BioTek Synergy™ 2 microplate reader. OriginPro 2021 9.8.0.200 was used for data processing.

Preparation of Tissue Sections

Fresh frozen NCI-H446 and NCI-H69 xenograft tumors were donated by the Research Group Imaging and Biomarkers in Oncology, TUM. The tumors were part of the referenced study,[2] and were harvested from eight-week-old athymic nude mice, Crl:NU/NCr-Foxn1nu, Charles River Laboratories, Sulzfeld. The mouse brain was harvested from an eight-week-old CD1 mouse for scientific purposes in accordance with the Tierschutzgesetz §7 Abs. 2 and provided by the Comparative Experimental Pathology, TUM. The tissues were embedded in HPMC/PVP-binary hydrogel (75:25) and cut into 10 µm thick sections using a Leica CM1950 cryostat (Leica Biosystems, Nussloch, Germany). The tissue sections were thaw-mounted onto Superfrost glass slides (epredia, Dreieich, Germany) and stored in a vacuum-packed stage at -80 °C until further use.

Mass Tag Staining Protocol

Tissue sections were rehydrated in cold PBS (4 °C) for 3 min. After the rehydration, the tissue sections were incubated with PARPi-MT PBS solution (0.2 mg/mL) for 45 min at r.t. in the dark. Subsequently, the tissue sections were washed in cold PBS (4 °C) with NAC (0.07 mg/mL) for 3 min, then in cold PBS (4 °C) for 2 x 3 min and dipped five times in cold aq. 1% TFA solution (4 °C). After washing, the tissue sections were dried under pressurized air and irradiated at 420 nm with a total radiant power of 2.9 W (93.75 mW/cm^2) for 30 min using a Lumidox® II LED controller and a 96-position single wavelength LED array (analytical, Flanders, US).

Desorption Electrospray Mass Spectrometry Imaging

DESI-MSI was performed on a Q Exactive™ Plus Hybrid Quadrupole-Orbitrap™ mass spectrometer (Thermo Fisher Scientific, Bremen, Germany). Images were recorded in positive ion mode with a mass range of 80–900 or 150–900 Da and a lateral resolution of 60 µm. Other settings were: 70,000 mass resolution at *m/z* 200, 250 ms injection time, 320 °C capillary temperature, and S-lens RF setting of 100. For the DESI-sprayer, the following settings were chosen: 1.5 mm sprayer-to-sample distance, 6 mm sprayer-to-inlet distance, 75° spray angle, 10° collection angle, 95% aq. methanol as spray solvent, 1.25 µL/min solvent flow rate, 4.5 kV spray voltage and 7 bar nebulizing gas pressure (N_2 purity N5.0). Individual line scans were

converted from the raw format to the imzML format using ProteoWizard MSConvert 3.0.23146-4ef0614,[3] and imzMLConverter.[4] MSiReader 0.06 (W.M. Keck Fourier Transform Mass Spectrometry Laboratory, NCSU, USA),[5] and LipostarMSI 2.1.0b5 (molecular Horizon srl., Bettona, Italy),[6] were used for data processing. TIC normalization was applied to all depicted images.

PARP1 Immunohistochemistry Protocol

The tissue sections were washed in PBS for 2 x 5 min and 1% (v/v) H_2O_2 in PBS was applied to the sections. After 30 min, the slides were washed in Millipore H_2O for 5 min and in PBS for 5 min. Subsequently, the tissue sections were incubated with 5% (v/v) goat serum (31873, Thermo Fisher Scientific, Darmstadt, Germany) in PBS at r.t. for 2 h. Afterwards, the slides were washed in PBS for 5 min and the sections were incubated in a 1:500 dilution of PARP1 polyclonal antibody (22999-1-AP, proteintech, Planegg-Martinsried, Germany) in PBS with 5% (w/v) BSA (A9418, Sigma-Aldrich, Saint Louis, USA) at r.t. for 2 h. Control sections were incubated with 5% (w/v) BSA in PBS at r.t. for 2 h. The slides were washed in PBS for 3 x 5 min and incubated in a 1:1000 dilution of goat anti-rabbit IgG (H+L) secondary antibody (65-6140, Thermo Fisher Scientific, Darmstadt, Germany) in PBS with 1% (w/v) BSA at r.t. for 10 min. Afterwards, the slides were washed in PBS for 5 min and 3 x 1 min. The slides were incubated in a 1:1000 dilution of streptavidin (ab7403, abcam, Cambridge, UK) in PBS with 1% (w/v) BSA at r.t. for 10 min. The slides were washed in PBS for 4 x 1 min. The washed slides were stained with DAB substrate kit (ab64238, abcam, Cambridge, UK) at r.t. for 1 min. Subsequently, the slides were washed in PBS for 3 x 1 min and stained with Mayers hematoxyline (3870.1000, J.T. Baker, Gliwice, Poland) at r.t. for 5 min. The slides were washed in cold tap water (4 °C) for 5 min, dipped in 50% (v/v) aq. EtOH 10 times, in 70% (v/v) aq. EtOH 10 times, washed in 95% (v/v) aq. EtOH for 30 s, in EtOH for 1 min and dipped in Neo-Clear™ five times. Finally, the slides were dried under pressurized air and cover slips were mounted on the tissue sections with DPX mountant (06522, Sigma-Aldrich, Taufkirchen, Germany). The slides were scanned using an Aperio AT slide scanner (Leica Biosystems, Wetzlar, Germany) with a resolution of 0.253 µm/pixel (corresponding to 40x magnification). Scans were processed with Aperio ImageScope 12.4.0.7018 (Leica Biosystems, Wetzlar, Germany).

Confocal Microscopy

After the DESI-MSI experiment, stained tissue sections were imaged using a Stellaris 5 confocal microscope (Leica Microsystems GmbH, Wetzlar, Germany) under the following conditions: X,Y = 0.6 µm pixel size, 20x magnification, 400 Hz scan speed, 405 nm excitation wavelength at 100% intensity, and detection within the 600-725 nm spectral range, with a gain setting of 30 and offset of 0. Image processing was performed using Leica Application Suite X 4.7.0.28176 (Leica Microsystems CMS GmbH, Mannheim, Germany).

Matrix Sprayer Instrumentation

Matrix application was performed using a HTX TM-Sprayer™ (HTX Technologies, LLC, Chapel Hill, USA), equipped with a heated spray nozzle and programmable XYZ motion controller for automated matrix deposition. The following parameters were used: 75 °C nozzle temperature, 40 mm nozzle height, 0.005 mL/min solvent flow rate, 1200 mm/min spray velocity, 10 psi N_2 gas pressure, 3 L/min N_2 gas flow rate, CC pattern, 2 mm track spacing, 10 passes and no drying time between passes. For the ion suppression control experiment by DESI-MSI, an aqueous solution of 10 µM [RuCl(phen)(tpy)]Cl and 10 µM TFA was used as the matrix.

Synthesis Protocols

General solvents and reagents were purchased from commercial suppliers and used without further purification if not stated otherwise. [RuCl(phen)(tpy)]Cl,[7] Biotin-PEG3-Boc,[8] and Biotin-PEG3-COOH,[9] were prepared as previously reported. Reactions involving Ru(II) complexes were conducted in the absence of light.

Olaparib-Biotin

4-(4-fluoro-3-(piperazine-1-carbonyl)benzyl)phthalazine-1-(2H)-one (315 mg, 860 µmol, 1.0 equiv.), D-biotin (234 mg, 958 µmol, 1.1 equiv.), DIPEA (358 µL, 272 mg, 2.11 mmol, 2.5 equiv.), and HCTU (396 mg, 957 µmol, 1.1 equiv.) were dissolved in DMF (8 mL). The reaction mixture was stirred at r.t. overnight. The reaction was quenched by the addition of H_2O (30 mL) and the solvent was removed under reduced pressure. The crude product was purified via flash chromatography (silica, DCM/MeOH, 8:2). The solvent mixture was removed under reduced pressure, affording Olaparib-biotin as a white solid in 91% yield (462 mg, 780 µmol).

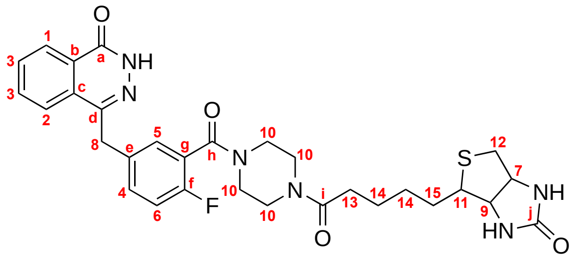

^{1}H-NMR (400 MHz, 294.6 K, CD_3OD) δ [ppm] = 8.37 (d, ^3J = 7.7 Hz, 1H, *H1*), 7.95 (d, ^3J = 7.9 Hz, 1H, *H2*), 7.85 (dt, ^3J = 16.1, 7.4 Hz, 2H, *H3*), 7.53–7.45 (m, 1H, *H4*), 7.40–7.34 (m, 1H, *H5*), 7.16 (t, ^3J = 9.0 Hz, 1H, *H6*), 4.49 (t, ^3J = 6.3 Hz, 1H, *H7*), 4.39 (s, 2H, *H8*), 4.31 (dd, ^3J = 8.0, 4.2 Hz, 1H, *H9*), 3.83–3.60 (m, 4H, *H10*), 3.56–3.42 (m, 2H, *H10*), 3.37–3.29 (m, 1H, *H10*), 3.25–3.17 (m, 1H, *H11*), 2.93 (dt, ^3J = 12.6, 4.5 Hz, 1H, *H12*), 2.70 (d, ^3J = 12.7 Hz, 1H, *H12*), 2.47 (t, ^3J = 7.3 Hz, 1H, *H13*), 2.40 (t, ^3J = 7.4 Hz, 1H, *H13*), 1.81–1.54 (m, 4H, *H14*), 1.54–1.40 (m, 2H, *H15*), 1.40–1.31 (m, 1H, *H10*).

^{13}C-NMR (100 MHz, 298.2 K, CD_3OD) δ [ppm] = 174.26 (*Ci*), 167.37 (R_2C=O), 162.29 (R_2C=O), 147.67 (*Cd*), 136.44 (*Ce*), 135.02 (*C3*), 133.46 (*Cg*), 133.37 (*C4*), 132.89 (*C3*), 130.90 (*Cc*), 130.12 (*C5*), 129.24 (*Cb*), 127.52 (*C1*), 126.78 (*C2*), 117.31 (*Cf*), 117.09 (*C6*), 63.35 (*C9*), 61.64 (*C7*), 57.00 (*C11*), 47.94 (*C10*), 46.28 (*C10*), 42.93 (*C10*), 42.24 (*C10*), 41.04 (*C12*), 38.19 (*C8*), 33.64 (*C13*), 29.83 (*C15*), 29.52 (*C14*), 26.23 (*C14*).

^{19}F-NMR (376 MHz, 298.1 K, CD_3OD) δ [ppm] = -120.74 (d, ^3J = 25.4 Hz, 1F).

HRMS: Calcd. for $C_{30}H_{33}FN_6O_4S$ $[M+Na]^+$: *m/z* = 615.2160; found: *m/z* = 615.2149.

Olaparib-PEG3-Biotin

4-(4-fluoro-3-(piperazine-1-carbonyl)benzyl)phthalazine-1-(2H)-one (90 mg, 246 µmol, 1.0 equiv.) and Biotin-PEG3-COOH (110 mg, 246 µmol, 1.0 equiv.) were dissolved in dry DCM/EtOH (5:1, 5 mL) and cooled to 0 °C. Under stirring, HOBt (37 mg, 270 µmol, 1.1 equiv.) and EDC · HCl (52 mg, 270 µmol, 1.1 equiv.) and DIPEA (86 µL, 64 mg, 494 µmol, 2.0 equiv.) were added. The reaction mixture was warmed up to r.t. and stirred at r.t. overnight. The solvent mixture was removed under reduced pressure. The crude product was purified via flash RP-chromatography (Biotage® Sfär C18 D) with a 10-90% gradient of eluent B (MeCN with 5% H_2O and 0.1% TFA) over 30 min (eluent A: H_2O with 0.1% TFA). The solvent mixture was removed under reduced pressure, affording Olaparib-PEG3-biotin as a colorless oil in 52% yield (102 mg, 128 µmol).

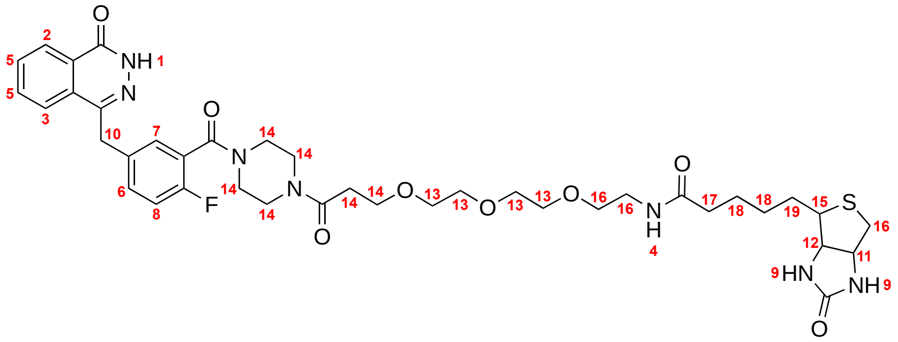

^{1}H-NMR (400 MHz, 298.1 K, DMSO-d_6) δ [ppm] = 12.60 (s, 1H, *H1*), 8.26 (d, ^3J = 7.8 Hz, 1H, *H2*), 7.96 (d, ^3J = 8.2 Hz, 1H, *H3*), 7.93–7.86 (m, 1H, *H4*), 7.86–7.78 (m, 2H, *H5*), 7.48–7.40 (m, 1H, *H6*), 7.40–7.31 (m, 1H, *H7*), 7.24 (t, ^3J = 8.9 Hz, 1H, *H8*), 6.59–6.14 (m, 2H, *H9*), 4.33 (s, 2H, *H10*), 4.30 (dd, ^3J = 7.8, 4.5 Hz, 1H, *H11*), 4.12 (dd, ^3J = 7.7, 4.4 Hz, 1H, *H12*), 3.68–3.57 (m, 8H, *H13*), 3.42–3.32 (m, 7H, *H14*), 3.21–3.11 (m, 4H, *H14*), 3.11–3.04 (m, 1H, *H15*), 2.81 (dd, ^3J = 12.5, 5.1 Hz, 1H, *H16*), 2.64–2.51 (m, 5H, *H16*), 2.05 (t, ^3J = 7.5 Hz, 2H, *H17*), 1.68–1.38 (m, 4H, *H18*), 1.38–1.18 (m, 2H, *H19*), 1.09 (t, ^3J = 7.0 Hz, 1H, *H14*).

^{19}F-NMR (471 MHz, 300.0 K, DMSO-d_6) δ [ppm] = -119.75 (dq, ^3J = 14.9, 6.7 Hz, 1F).

HRMS: Calcd. for $C_{39}H_{50}FN_7O_8S$ $[M+Na]^+$: *m/z* = 818.3318; found: *m/z* = 818.3293.

PARPi-MT – [Ru(Olaparib-biotin)(phen)(tpy)]Cl$_2$

[RuCl(phen)(tpy)]Cl (200 mg, 342 µmol, 1.0 equiv.) and Olaparib-Biotin (223 mg, 376 µmol, 1.1 equiv.) were suspended in Millipore H_2O (15 ml). The suspension was heated at 110 °C in a microwave reactor overnight. The crude product was obtained via lyophilization and purified via flash chromatography (Biotage® Sfär Silica D) with a 1-10% gradient of eluent B (1 M aq. KCl) over 20 min (eluent A: MeOH). The solvent mixture was removed under reduced pressure at 30 °C. The crude product was extracted with cold EtOH (-80 °C), and an excess of acetone was added to precipitate the product. The precipitate was washed with Et_2O (1 x 20 ml) and dried *in vacuo*, affording PARPi-MT as an orange powder in 46% yield (189 mg, 160 µmol).

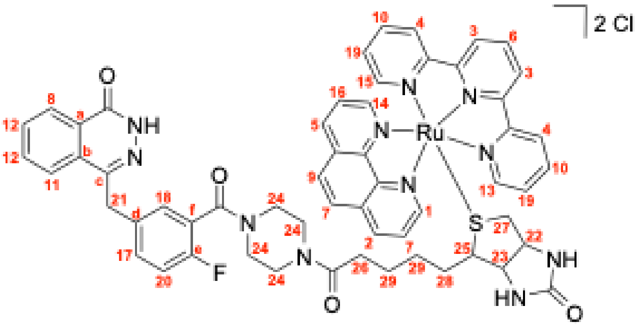

^{1}H-NMR (400 MHz, 298.2 K, CD_3OD) δ [ppm] = 10.18 (d, ^3J = 5.2 Hz, 1H, *H1*), 9.02 (d, ^3J = 8.2 Hz, 1H, *H2*), 8.90 (dd, ^3J = 13.9, 8.1 Hz, 2H, *H3*), 8.72 (dd, ^3J = 19.4, 8.1 Hz, 2H, *H4*), 8.56 (d, ^3J = 8.1 Hz, 1H, *H5*), 8.48 (t, ^3J = 8.1 Hz, 1H, *H6*), 8.42 (d, ^3J = 8.9 Hz, 2H, *H7*), 8.35 (t, ^3J = 6.5 Hz, 1H, *H8*), 8.24 (d, ^3J = 8.8 Hz, 1H, *H9*), 8.08 (t, ^3J = 7.7 Hz, 2H, *H10*), 7.96 (d, ^3J = 8.0 Hz, 1H, *H11*), 7.85 (dt, ^3J = 22.0, 7.3 Hz, 2H, *H12*), 7.76 (d, ^3J = 5.5 Hz, 1H, *H13*), 7.68 (d, ^3J = 5.3 Hz, 1H, *H14*), 7.64 (d, ^3J = 5.5 Hz, 1H, *H15*), 7.59 (dd, ^3J = 8.2, 5.3 Hz, 1H, *H16*), 7.56–7.46 (m, 1H, *H17*), 7.41–7.34 (m, 1H, *H18*), 7.32 (t, ^3J = 6.7 Hz, 2H, *H19*), 7.17 (t, ^3J = 9.0 Hz, 1H, *H20*), 4.39 (s, 2H, *H21*), 4.28–4.22 (m, 1H, *H22*), 4.22–4.16 (m, 1H, *H23*), 3.85–3.74 (m, 1H, *H24*), 3.74–3.69 (m, 1H, *H24*), 3.69–3.63 (m, 1H, *H24*), 3.63–3.56 (m, 1H, *H24*), 3.54–3.45 (m, 1H, *H24*), 3.45–3.37 (m, 1H, *H24*), 3.30–3.24 (m, 1H, *H24*), 2.50–2.41 (m, 1H, *H25*), 2.37–2.19 (m, 2H, *H26*), 2.05 (d, ^3J = 12.2 Hz, 1H, *H27*), 1.91 (dd, ^3J = 12.2, 4.8 Hz, 1H, *H27*), 1.57–1.42 (m, 1H, *H28*), 1.40–1.26 (m, 1H, *H29*), 1.26–1.15 (m, 2H, *H29*), 1.15–1.04 (m, 1H, *H24*), 0.74–0.59 (m, 1H, *H29*), 0.59–0.46 (m, 1H, *H28*).

^{13}C-NMR (100 MHz, 300.3 K, CD_3OD) δ [ppm] = 167.36 (*Cf*), 159.66 (R_2C=O), 159.45 (R_2C=O), 158.82 (R_2C=O), 158.78 (R_2C=O), 154.96 (*C13*), 154.52 (*C15*), 154.06 (*C1*), 151.62 (*C14*), 148.74 (*Ca*), 148.18 (*Cc*), 147.70 (*Cb*), 140.39 (*C10*), 138.93 (*C2*), 138.81 (*C5*), 138.68 (*C6*), 136.45 (*Ce*), 135.04 (*C12*), 133.45 (*C17*), 132.89 (*C12*), 132.20 (*Cd*), 130.09 (*C18*), 129.73 (*C19*), 129.56 (*C7*), 129.09 (*C9*), 127.71 (*C7*), 127.49 (*C8*), 126.93 (*C16*), 126.69 (*C11*), 126.51 (*C4*), 125.95 (*C3*), 117.10 (*C20*), 60.37 (*C23*), 58.66 (*C22*), 57.68 (*C25*), 46.65 (*C24*),

43.29 (*C24*), 42.87 (*C24*), 42.51 (*C24*), 40.92 (*C27*), 38.17 (*C21*), 33.22 (*C26*), 28.27 (*C28*), 27.89 (*C29*), 25.55 (*C29*).

^{19}F-NMR (376 MHz, 298.1 K, CD_3OD) δ [ppm] = -120.75 (d, ^3J = 20.1 Hz, 1F).

HRMS: Calcd. For $C_{57}H_{52}FN_{11}O_4RuS$ $[M]^{2+}$: *m/z* = 553.6471; found: *m/z* = 553.6469.

PARPi-PEG3-MT – [Ru(Olaparib-PEG3-biotin)(phen)(tpy)]Cl$_2$

[RuCl(phen)(tpy)]Cl (70 mg, 120 µmol, 1.0 equiv.) and Olaparib-PEG3-biotin (124 mg, 156 µmol, 1.3 equiv.) were suspended in Millipore H_2O (12 ml). The suspension was heated at 100 °C in a microwave reactor for 5 h. The crude product was obtained via lyophilization and purified via semi-preparative RP-HPLC with a 10-90% gradient of eluent B (MeCN) over 20 min (eluent A: H_2O). MeCN was removed under reduced pressure at 30 °C and the concentrated solution was lyophilized, affording PARPi-PEG3-MT as an orange oil in 9% yield (14 mg, 11 µmol).

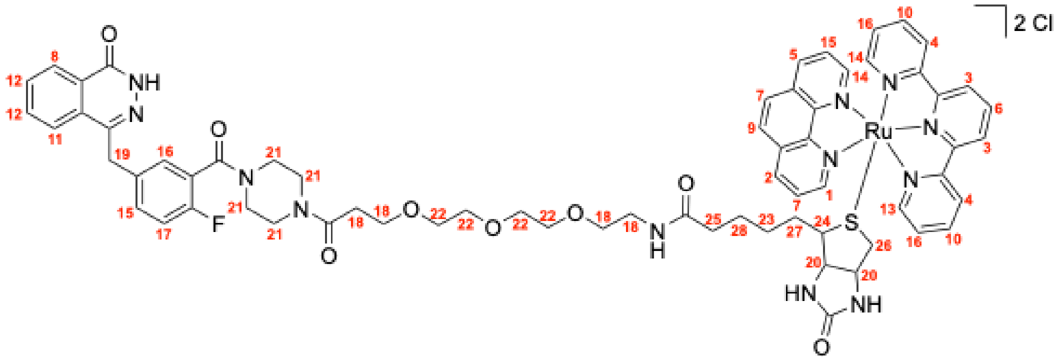

^{1}H-NMR (400 MHz, 296.3 K, CD_3OD) δ [ppm] =10.17 (d, ^3J = 5.2 Hz, 1H, *H1*), 9.01 (d, ^3J = 8.0 Hz, 1H, *H2*), 8.93–8.82 (m, 2H, *H3*), 8.75–8.63 (m, 2H, *H4*), 8.55 (d, ^3J = 8.0 Hz, 1H, *H5*), 8.48 (d, ^3J = 8.0 Hz, 1H, *H6*), 8.46–8.38 (m, 2H, *H7*), 8.38–8.33 (m, 1H, *H8*), 8.23 (d, ^3J = 8.8 Hz, 1H, *H9*), 8.08 (d, ^3J = 7.2 Hz, 2H, *H10*), 7.96 (d, ^3J = 7.9 Hz, 1H, *H11*), 7.92–7.83 (m, 2H, *H12*), 7.76 (d, ^3J = 5.8 Hz, 1H, *H13*), 7.69–7.60 (m, 2H, *H14*), 7.60–7.52 (m, 2H, *H15*), 7.36–7.29 (m, 3H, *H16*), 7.22–7.16 (m, 1H, *H17*), 5.49 (s, 4H, *H18*), 4.39 (s, 2H, *H19*), 4.23–4.12 (m, 2H, *H20*), 3.81–3.63 (m, 5H, *H21*), 3.63–3.56 (m, 4H, *H18*), 3.56–3.43 (m, 8H, *H22*), 3.40–3.28 (m, 2H, *H21*), 2.69 (t, ^3J = 6.1 Hz, 1H, *H21*), 2.66–2.58 (m, 1H, *H23*), 2.44–2.36 (m, 1H, *H24*), 2.35 (s, 1H, *H23*), 2.08–2.01 (m, 2H, *H25*), 2.00 (s, 1H, *H26*), 1.90–1.82 (m, 1H, *H26*), 1.56–1.27 (m, 1H, *H27*), 1.24–1.04 (m, 2H, *H28*), 0.68–0.47 (m, 1H, *H27*).

^{19}F-NMR (471 MHz, 298.0 K, CD_3OD) δ [ppm] = -120.63 (d, ^3J = 25.4 Hz, 1F).

HRMS: Calcd. for $C_{66}H_{69}FN_{12}O_8RuS$ $[M]^{2+}$: *m/z* = 655.2049; found: *m/z* = 655.2023.

Spectra

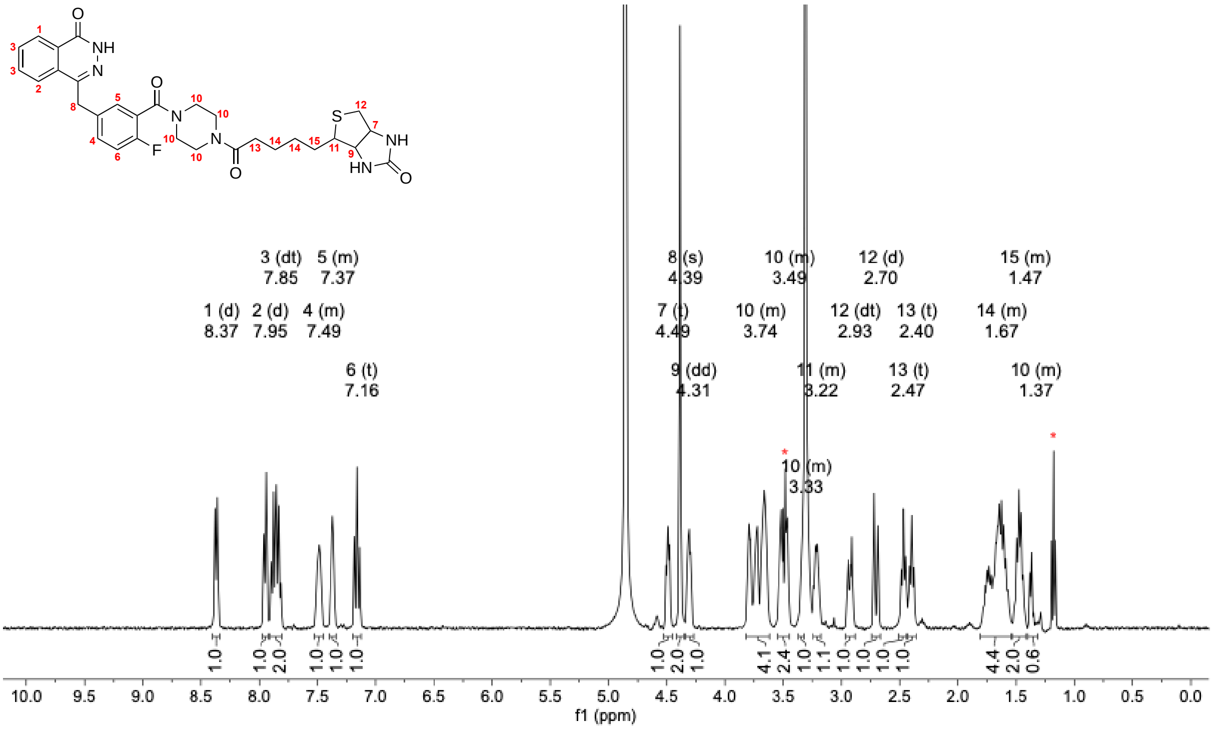

Figure S1. ^{1}H NMR spectrum of **Olaparib-Biotin** (400 MHz, CD_3OD). Solvent impurities (Et_2O) are marked with a red asterisk.

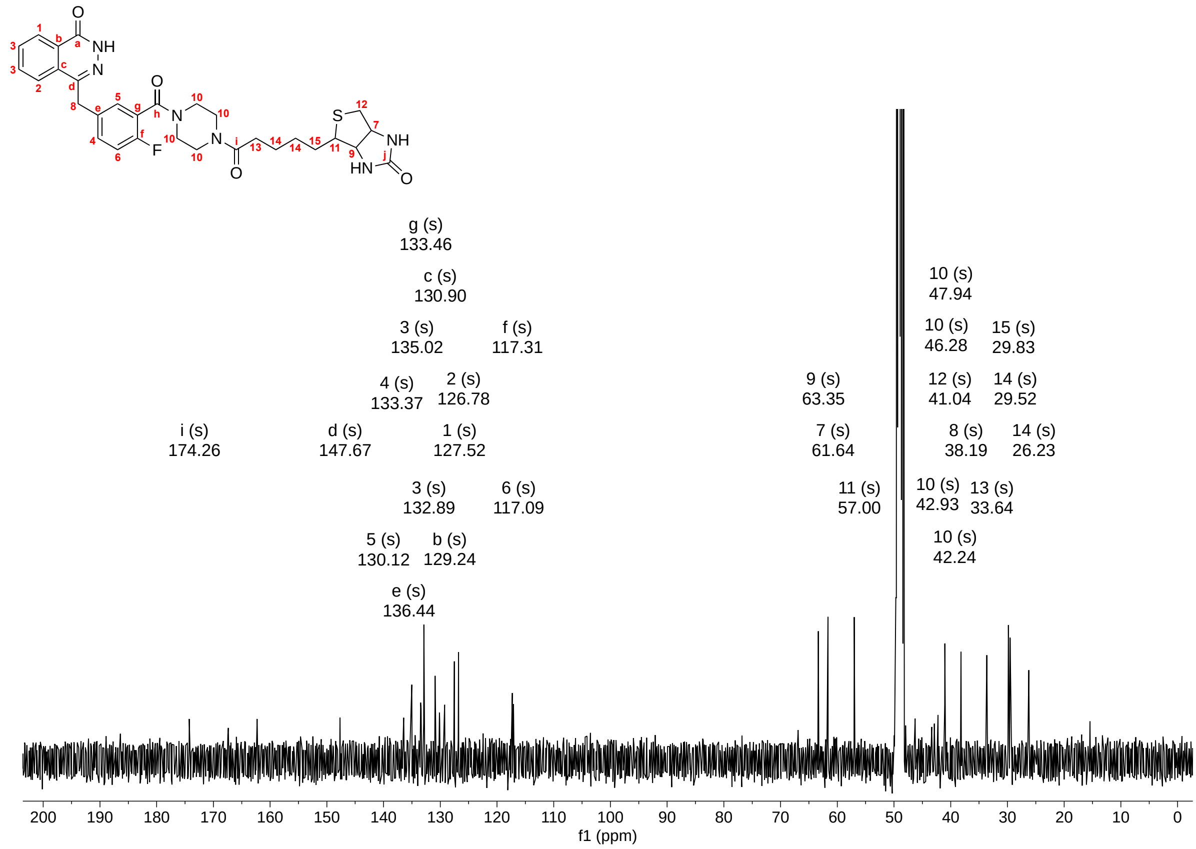

Figure S2. ^{13}C NMR spectrum of **Olaparib-biotin** (100 MHz, CD_3OD).

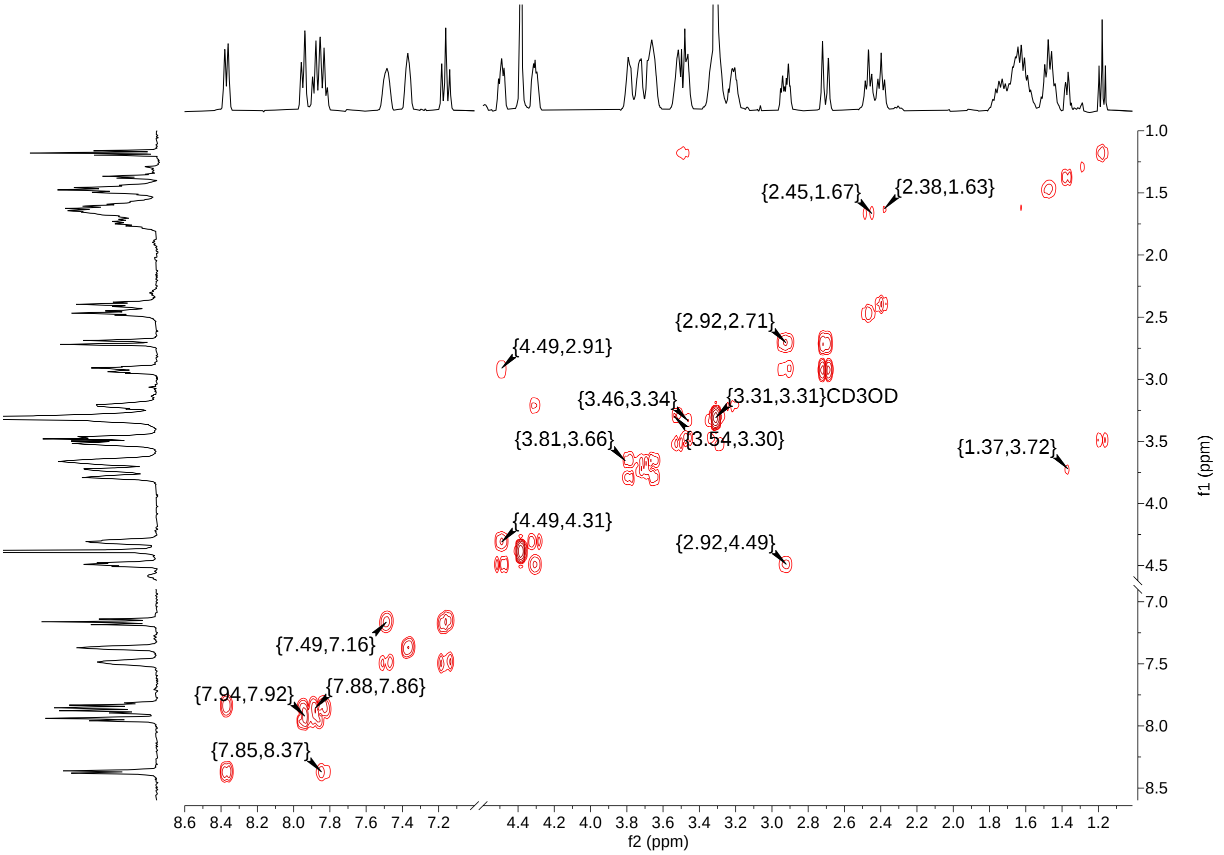

Figure S3. ^{1}H,^{1}H-COSY NMR spectrum of **Olaparib-biotin** in CD_3OD.

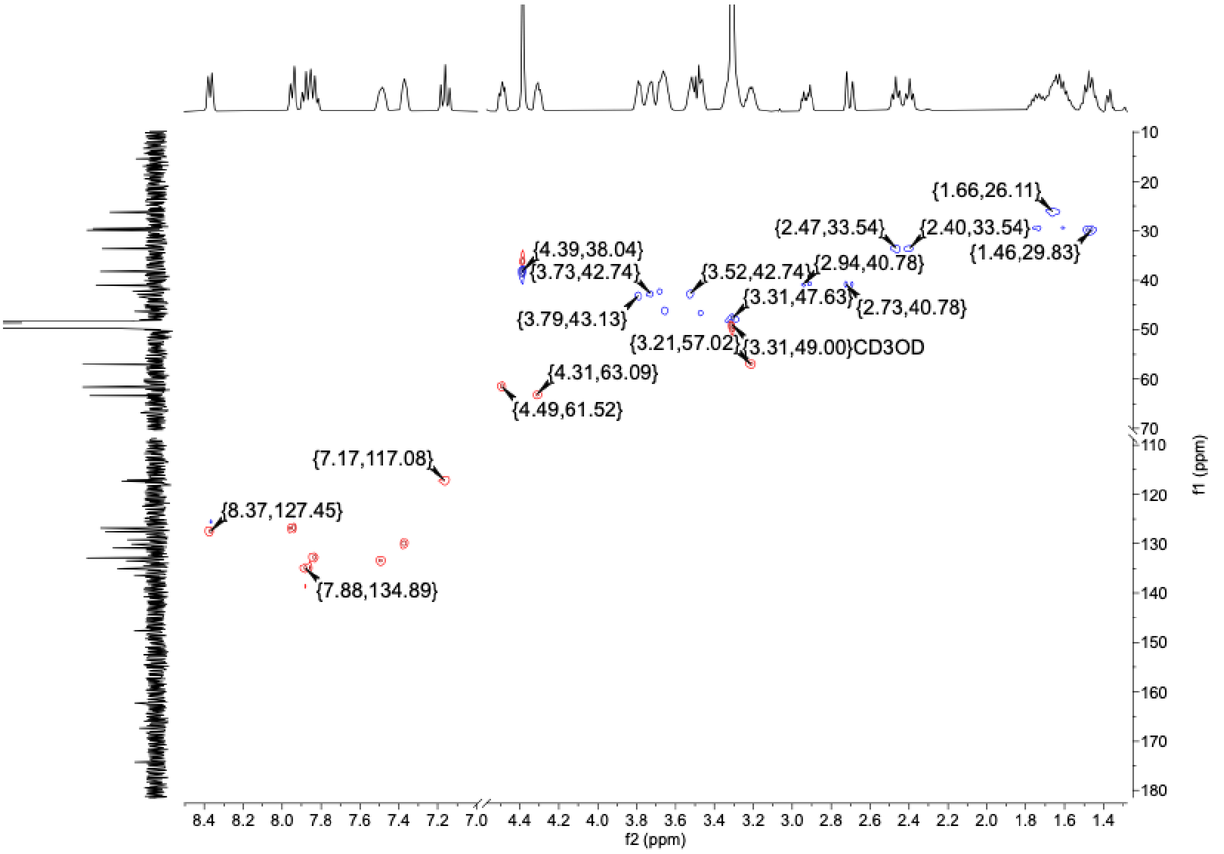

Figure S4. ^{1}H,^{13}C-HSQC spectrum of **Olaparib-biotin** in CD_3OD.

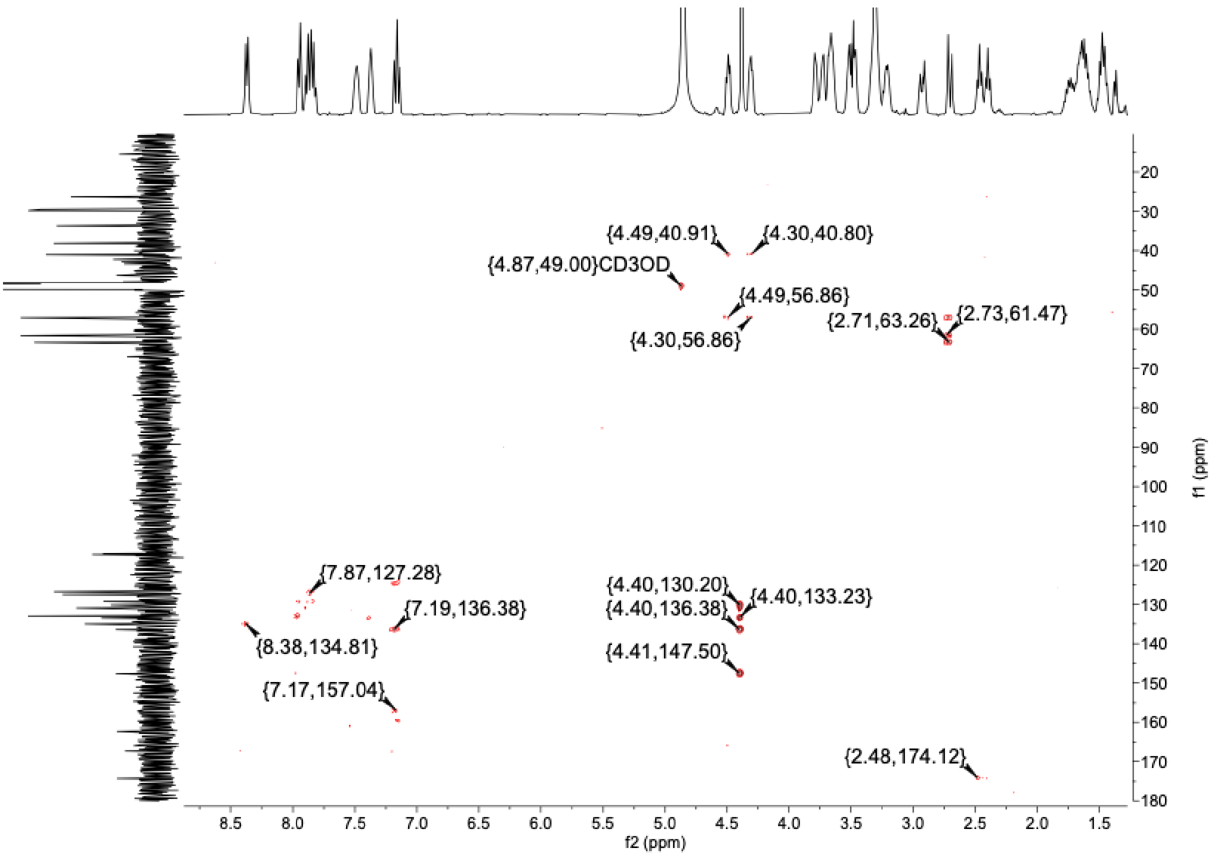

Figure S5. ^{1}H,^{13}C-HMBC spectrum of **Olaparib-biotin** in CD_3OD.

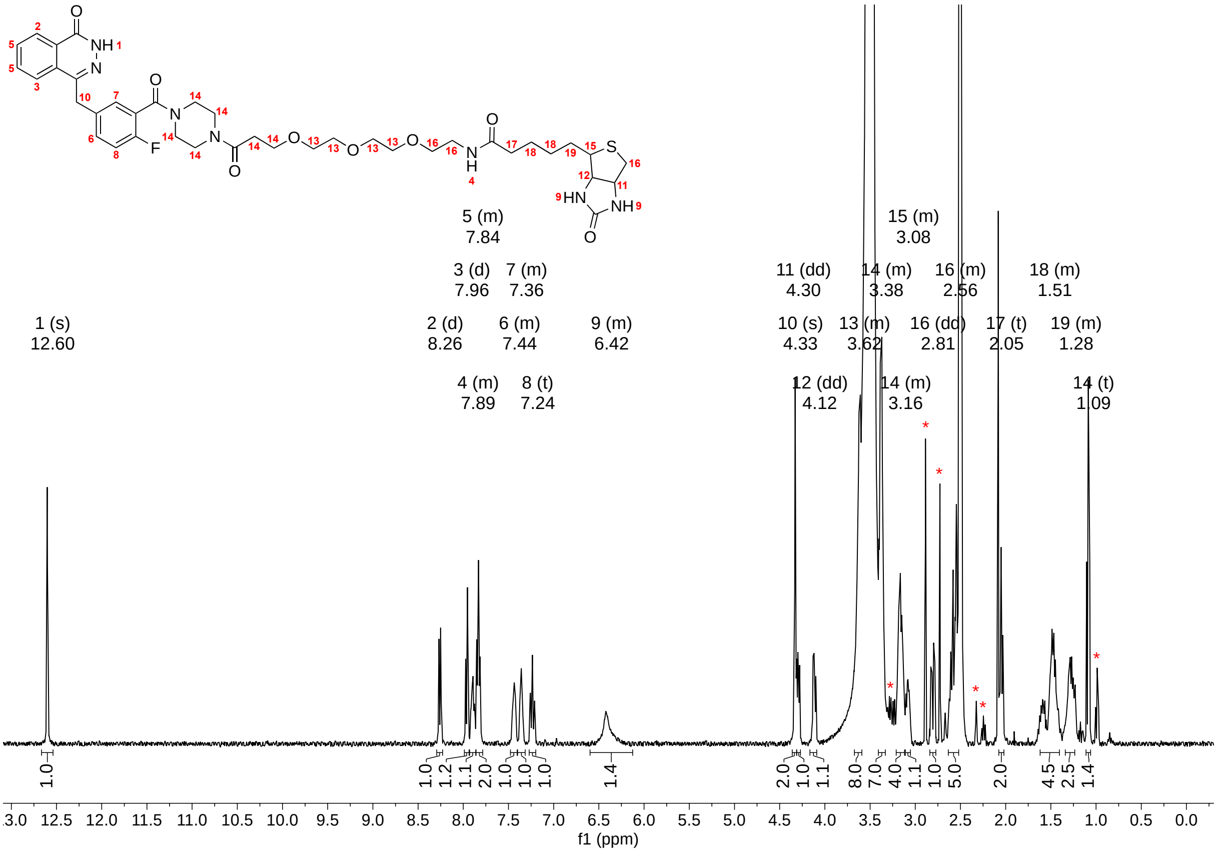

Figure S6. ^{1}H NMR spectrum of **Olaparib-PEG3-biotin** (400 MHz, DMSO-d_6). Solvent impurities (DMF, MeCN, EtOH) are marked with a red asterisk.

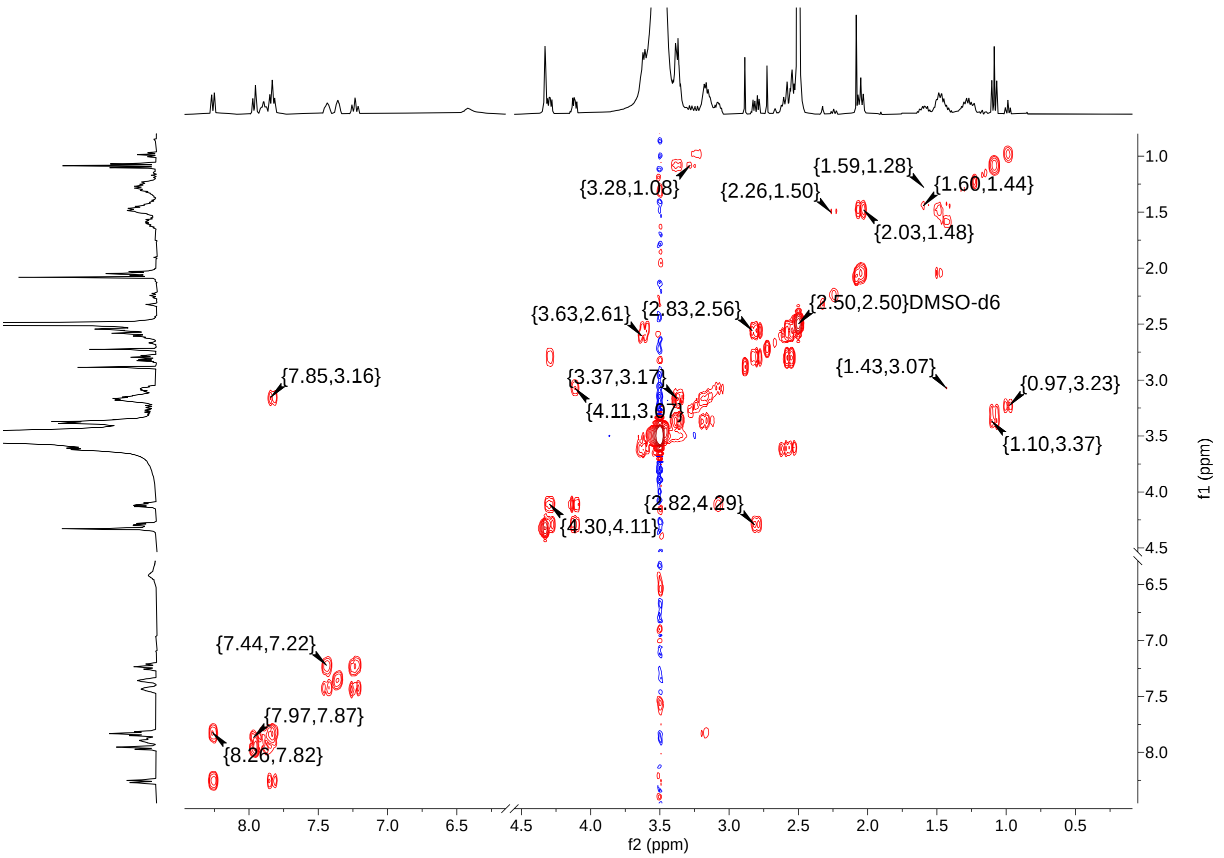

Figure S7. $^{1}H,^{1}H$-COSY NMR spectrum of **Olaparib-PEG3-biotin** in DMSO-d_6.

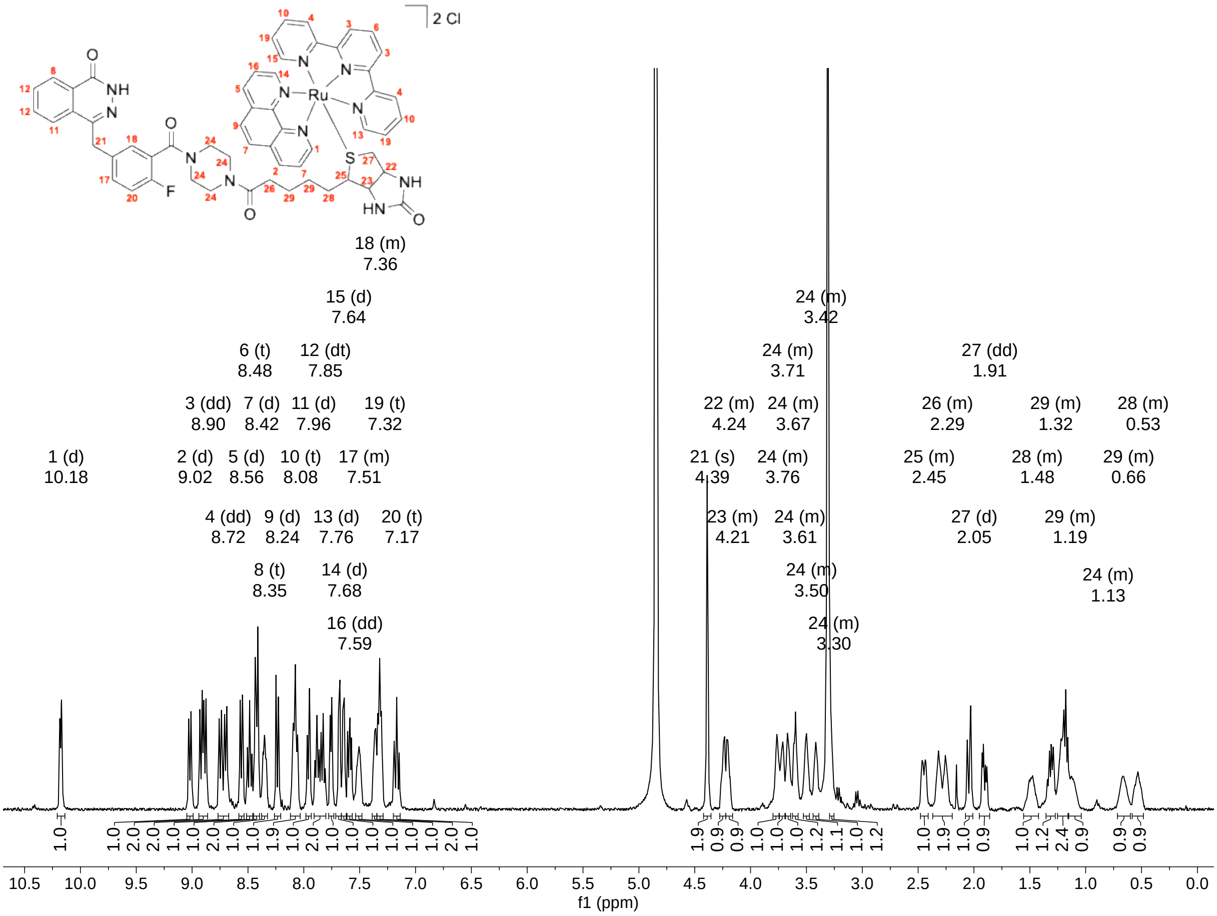

Figure S8. ^{1}H NMR spectrum of **PARPi-MT** (400 MHz, CD_3OD).

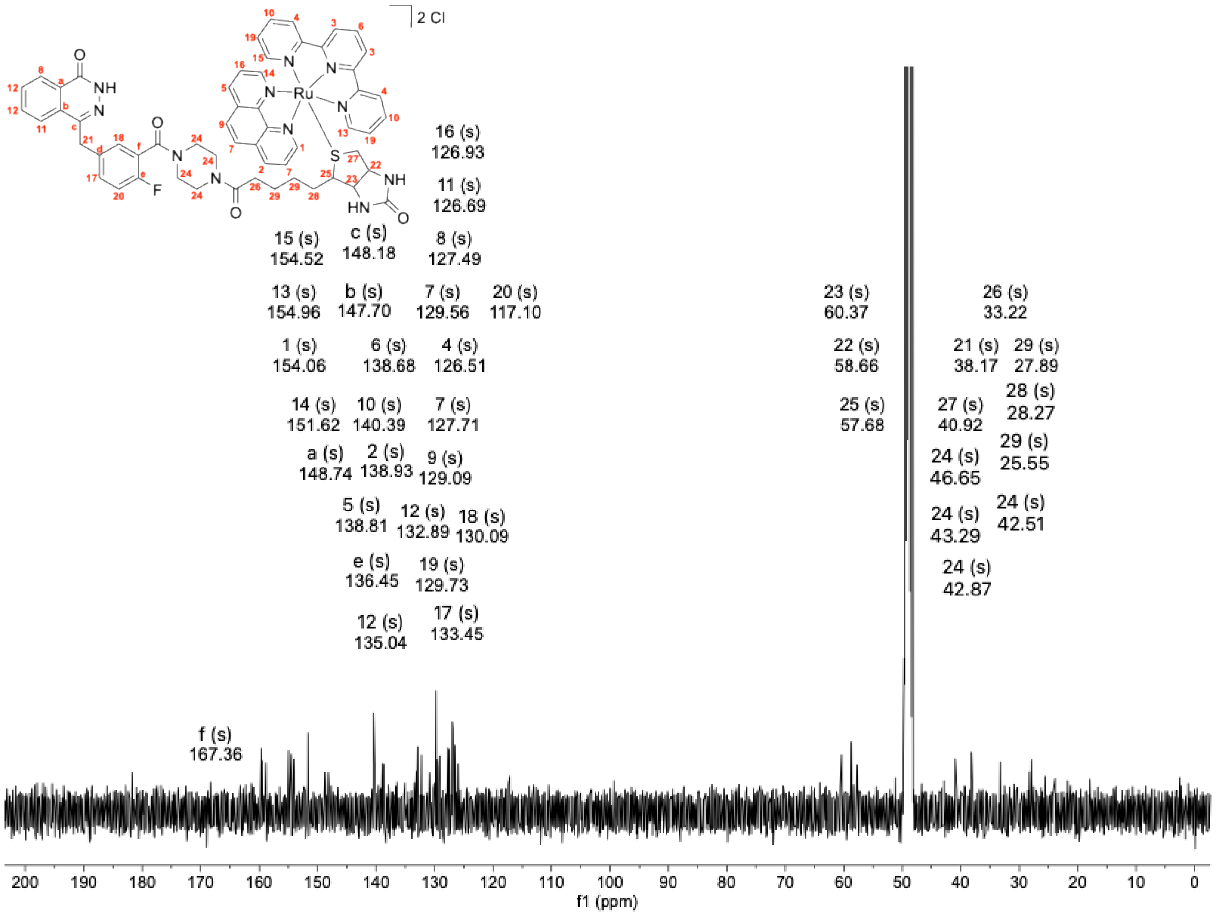

Figure S9. ^{13}C NMR spectrum of **PARPi-MT** (100 MHz, CD_3OD).

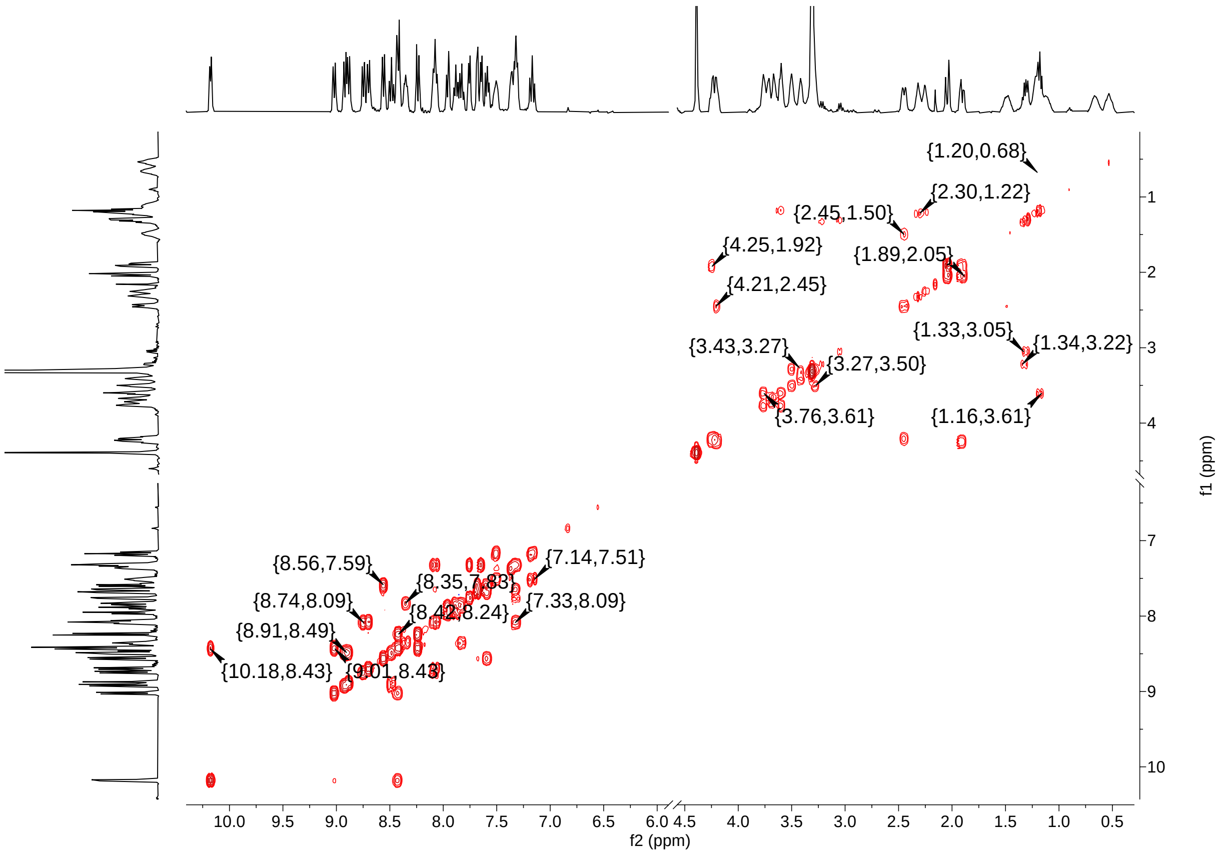

Figure S10. $^{1}H,^{1}H$-COSY NMR spectrum of **PARPi-MT** in CD_3OD.

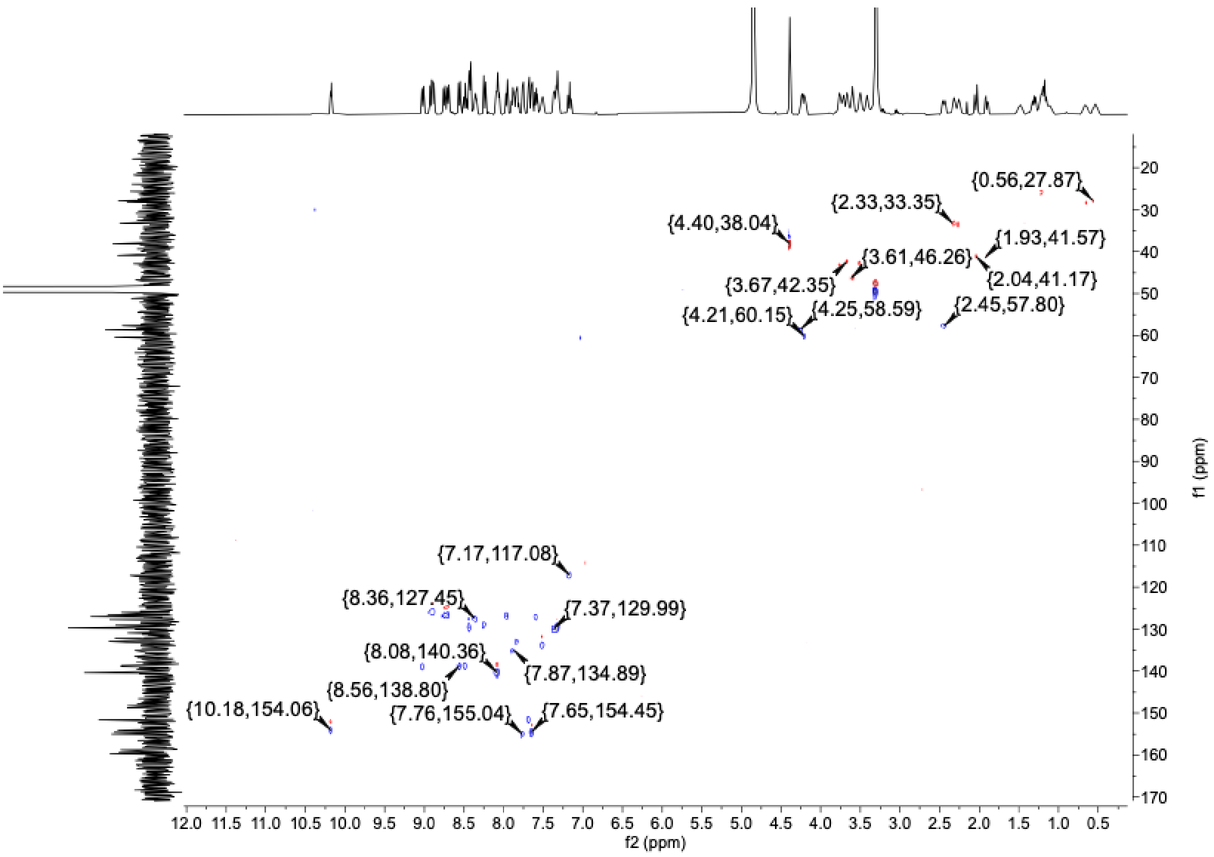

Figure S11. $^{1}H,^{13}C$-HSQC spectrum of **PARPi-MT** in CD_3OD.

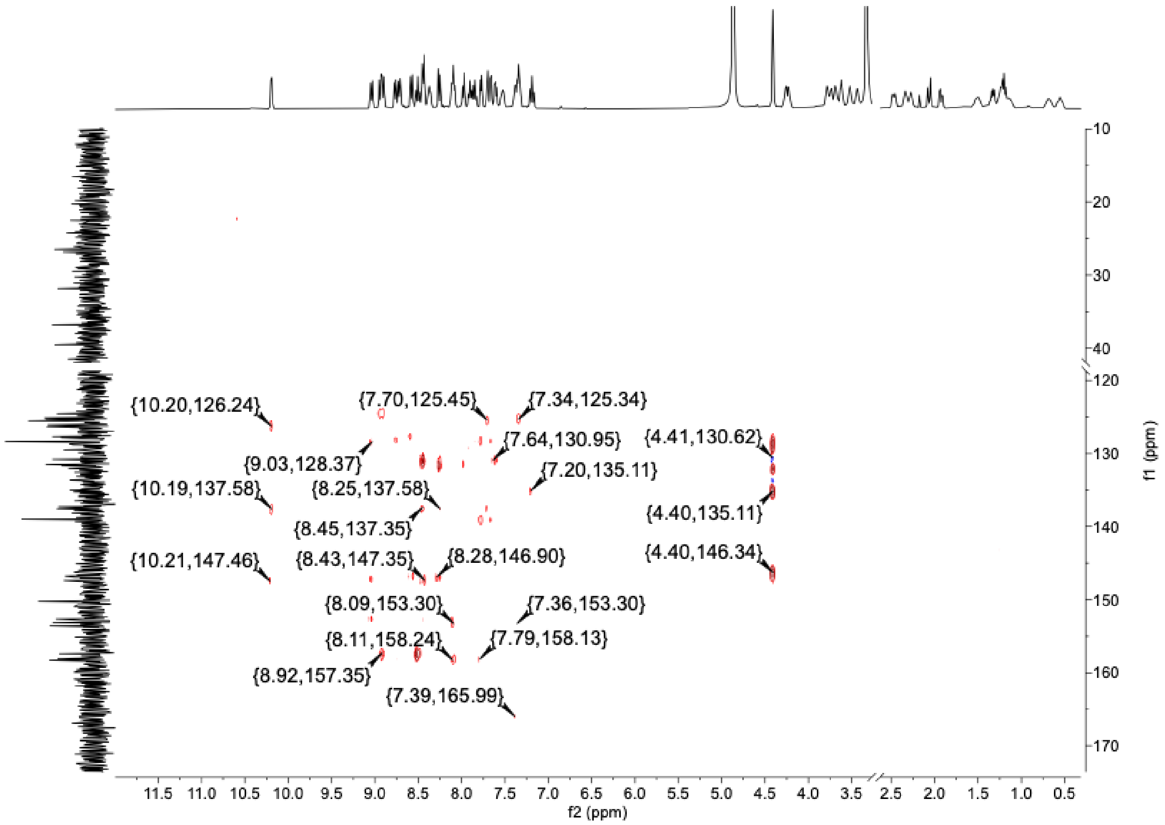

Figure S12. $^{1}H,^{13}C$ HMBC spectrum of **PARPi-MT** in CD_3OD.

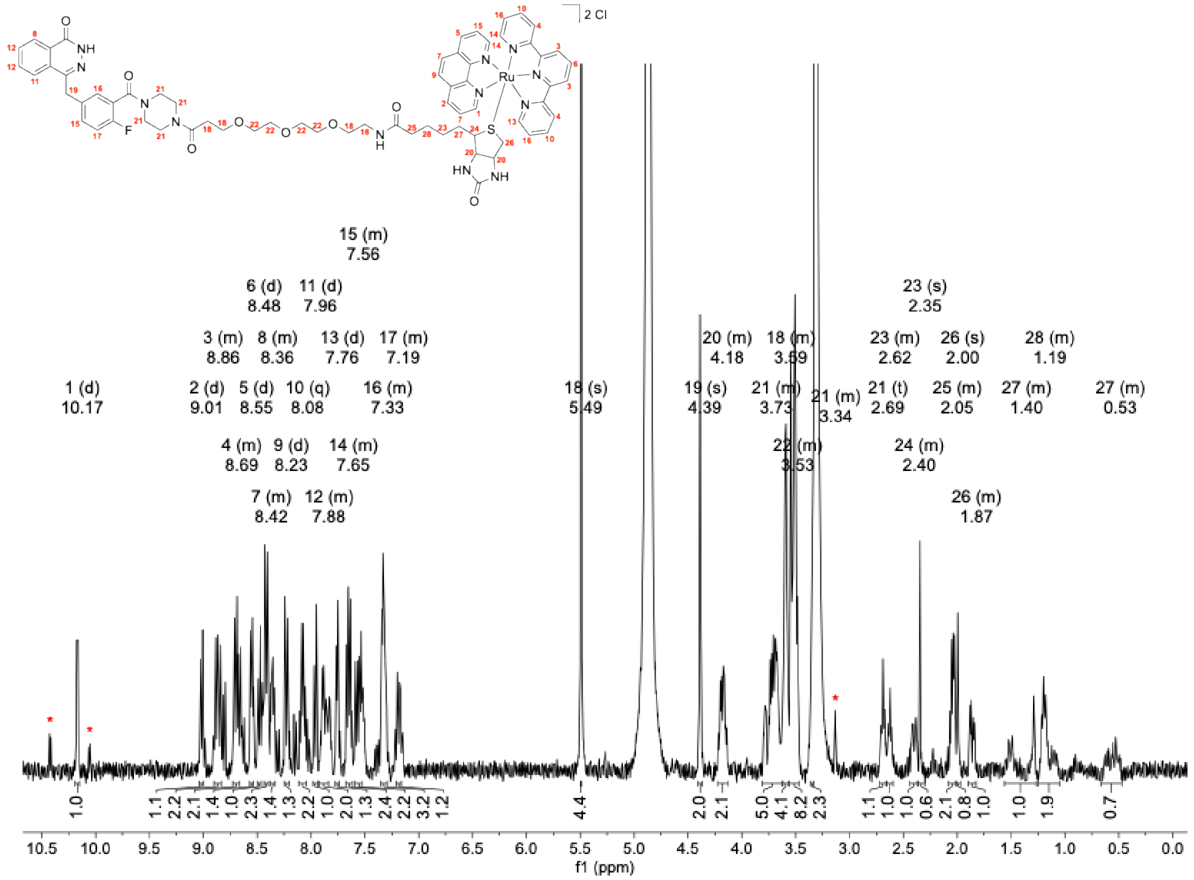

Figure S13. ^1H NMR spectrum of **PARPi-PEG3-MT** (400 MHz, CD_3OD). Impurities are marked with a red asterisk.

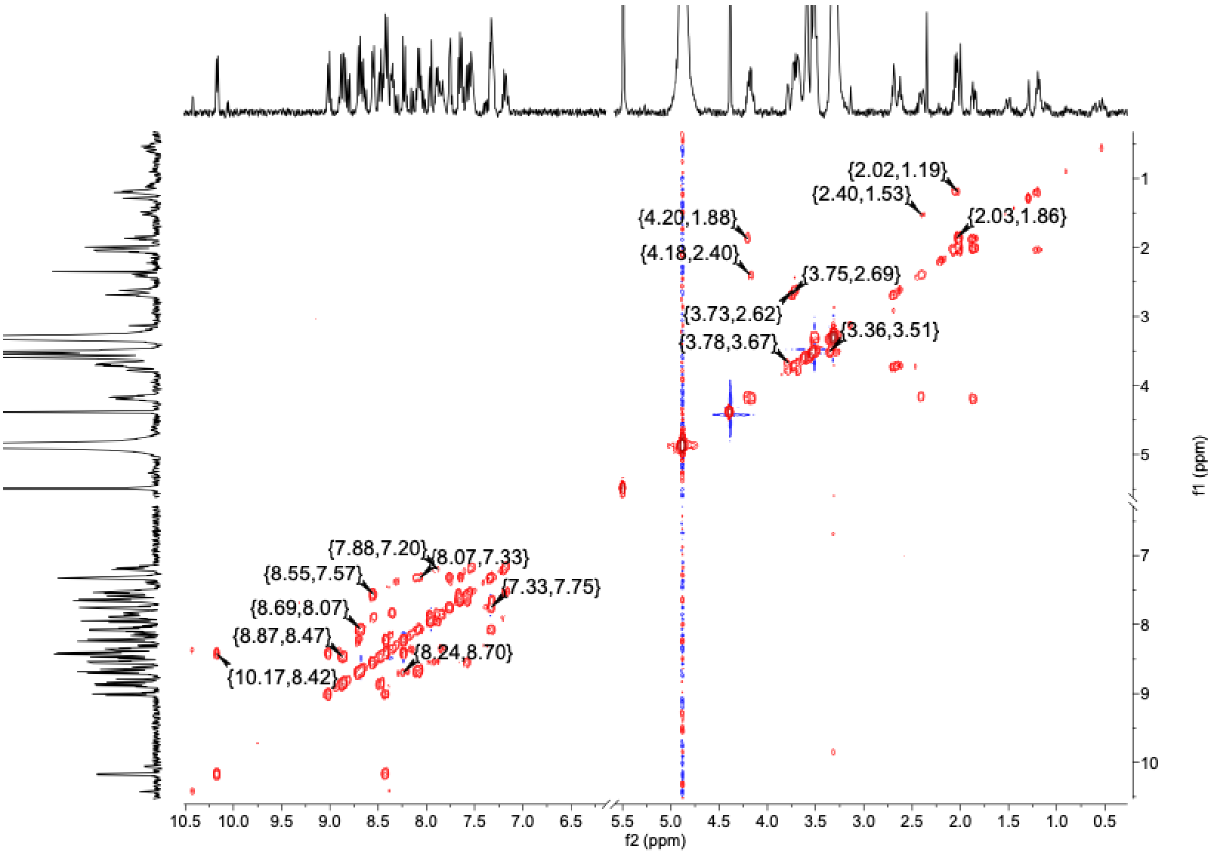

Figure S14. ^1H,^1H-COSY NMR spectrum of **PARPi-PEG3-MT** in CD_3OD.

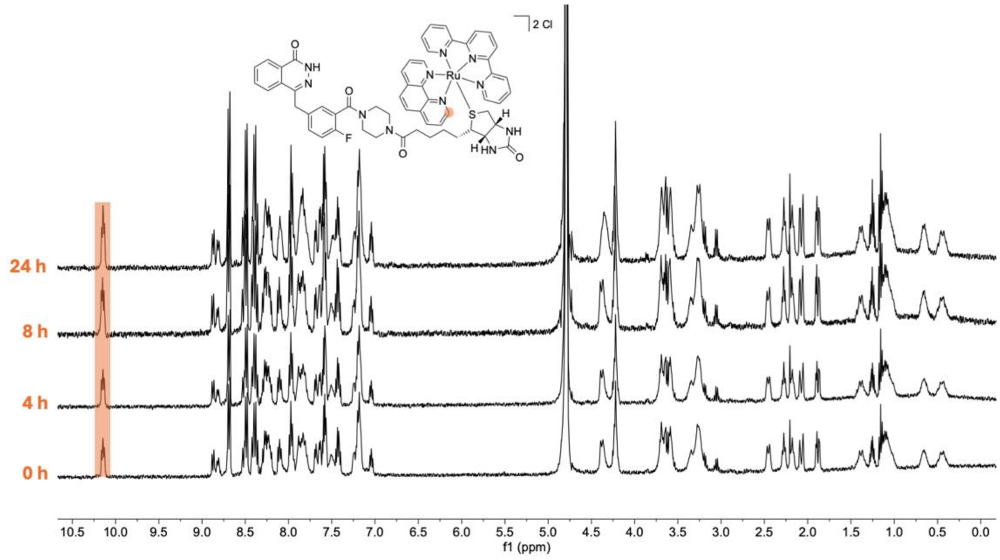

Figure S15. The stability study of **PARPi-MT** in D_2O was conducted by recording ^{1}H NMR spectra of the solution (3.5 µM) at defined time intervals (t = 0, 4, 8, 24 h). The signal corresponding to the marked proton on the 1,10-phenanthroline ligand was monitored. No change in peak integral or spectral profile was observed, indicating that **PARPi-MT** remains stable in D_2O under ambient conditions.

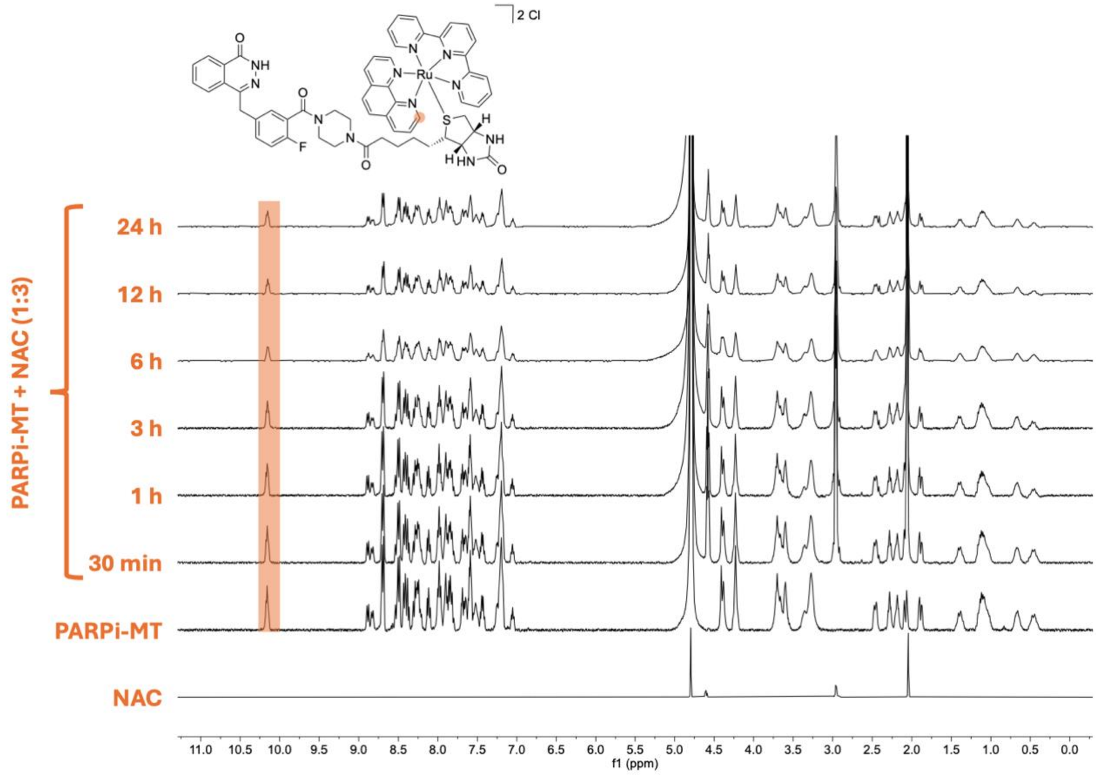

Figure S16. The stability of **PARPi-MT** in D_2O in the presence of NAC was assessed. To a solution of **PARPi-MT** in D_2O (3.5 mM, 300 µL), 10 µL of a NAC solution (300 mM) in D_2O was added, resulting in a final mixture with **PARPi-MT** and NAC at a 1:3 molar ratio. The signal corresponding to the marked proton on the 1,10-phenanthroline ligand was monitored at defined time intervals (t = 30 min, 1, 3, 6, 12, 24 h) over a period of 24 h. No change in peak integral or spectral profile was observed, indicating that **PARPi-MT** remains stable in the presence of NAC under ambient conditions.

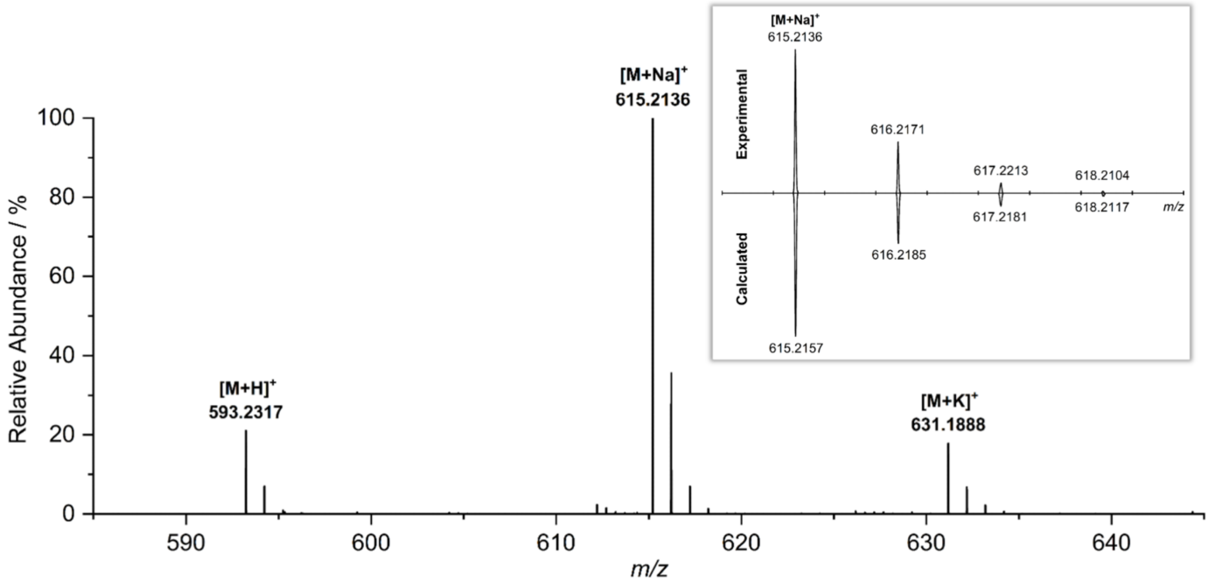

Figure S17. HESI-MS spectrum of **Olaparib-Biotin** ($C_{30}H_{33}FN_6O_4S$) in LC-MS grade MeOH.

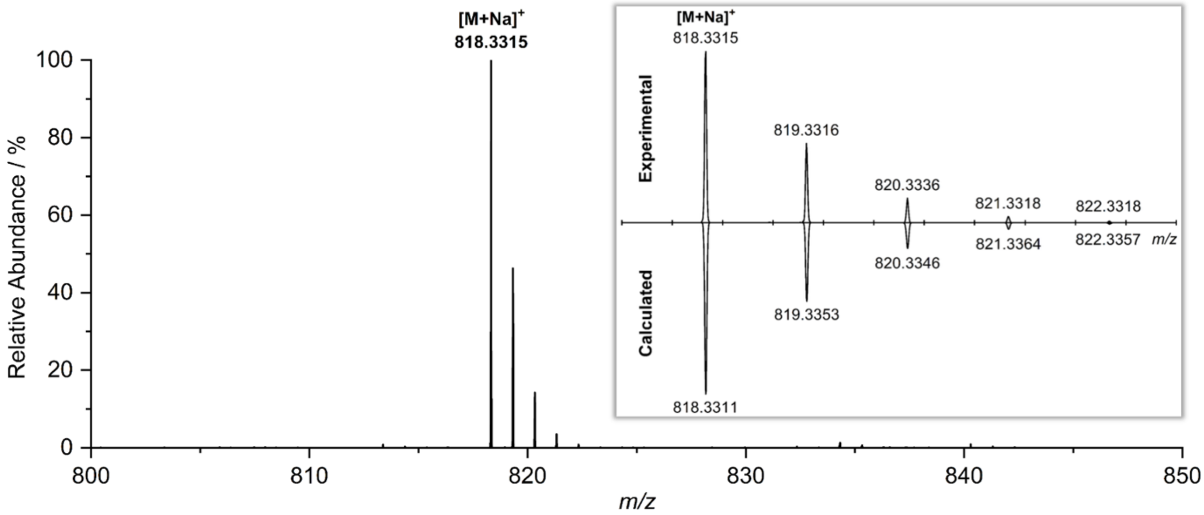

Figure S18. HESI-MS spectrum of **Olaparib-PEG3-Biotin** ($C_{39}H_{50}FN_7O_8S$) in LC-MS grade MeCN.

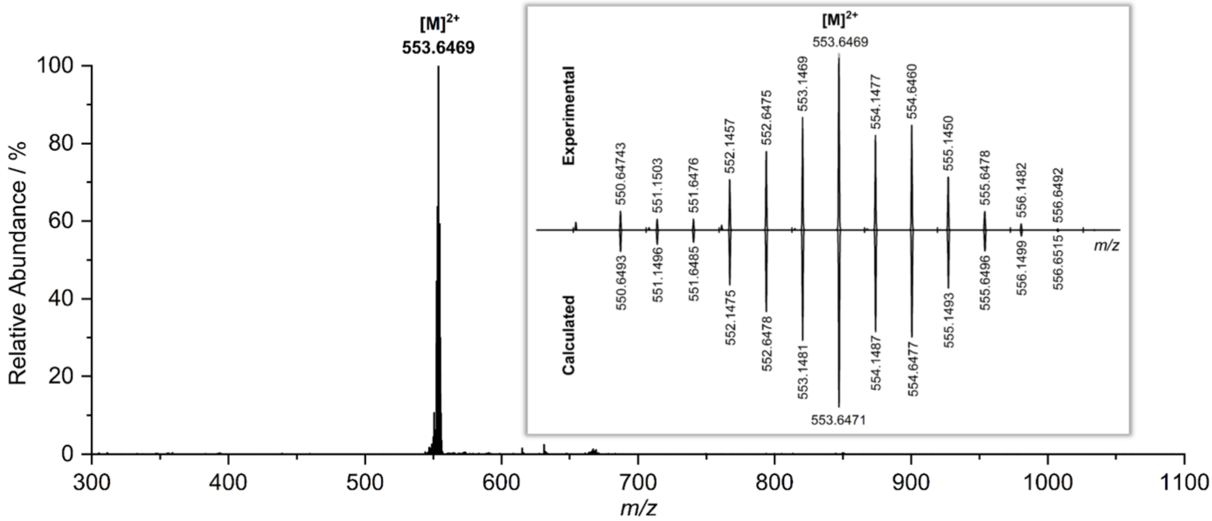

Figure S19. HESI-MS spectrum of **PARPi-MT** ($C_{57}H_{52}FN_{11}O_4RuS$) in LC-MS grade MeOH.

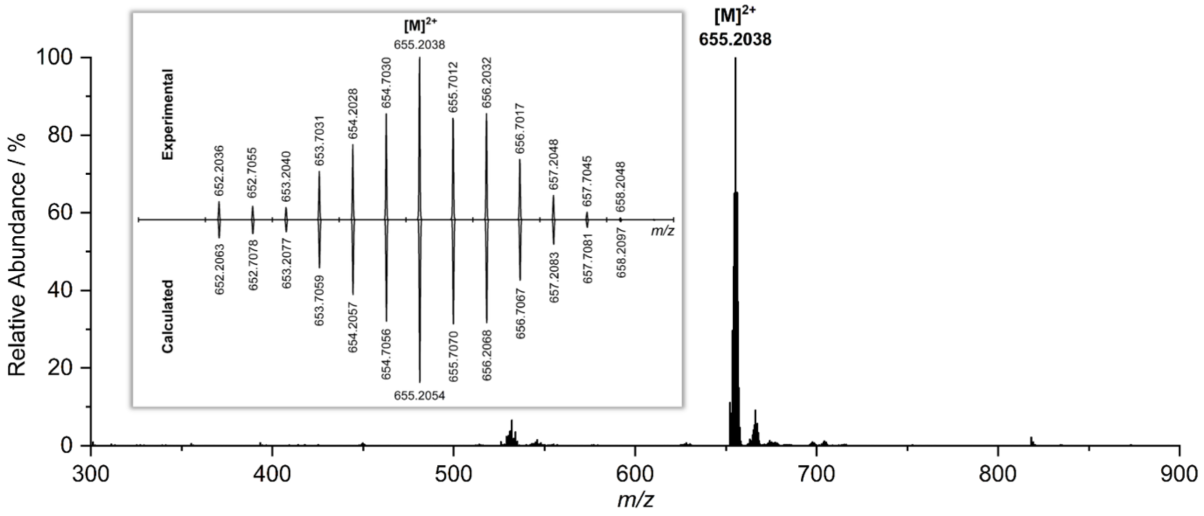

Figure S20. HESI-MS spectrum of **PARPi-PEG3-MT** ($C_{66}H_{69}FN_{12}O_8RuS$) in LC-MS grade MeOH.

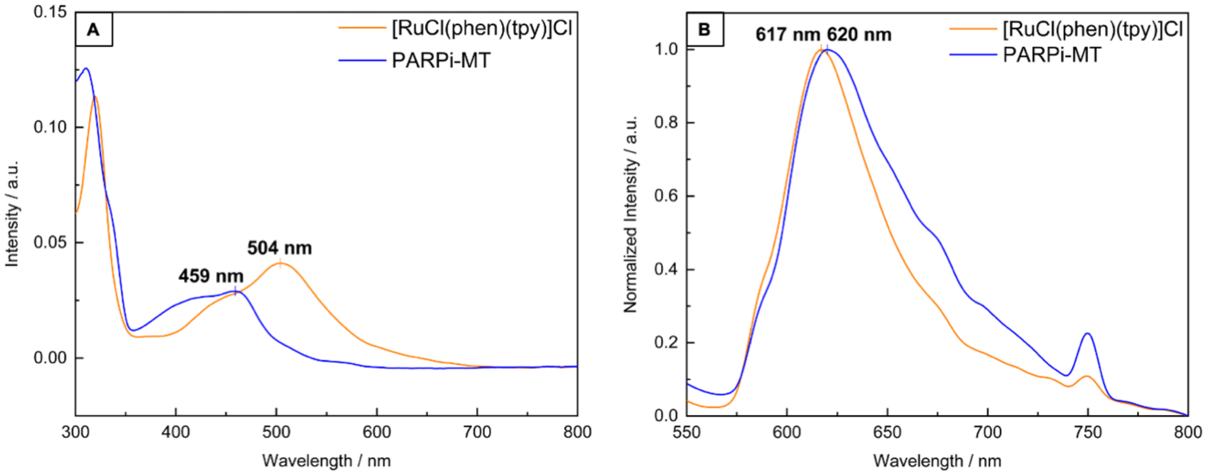

Figure S21. A: UV-Vis absorption spectra of [RuCl(phen)(tpy)]Cl (orange trace) and **PARPi-MT** (blue trace) in THF (5 µM). B: Normalized emission spectra (λ_{ex} = 375 nm) of [RuCl(phen)(tpy)]Cl (orange trace) and **PARPi-MT** (blue trace) in Ar-saturated THF (5 µM). The signal at λ_{em} = 750 nm corresponds to the second harmonic of the excitation.

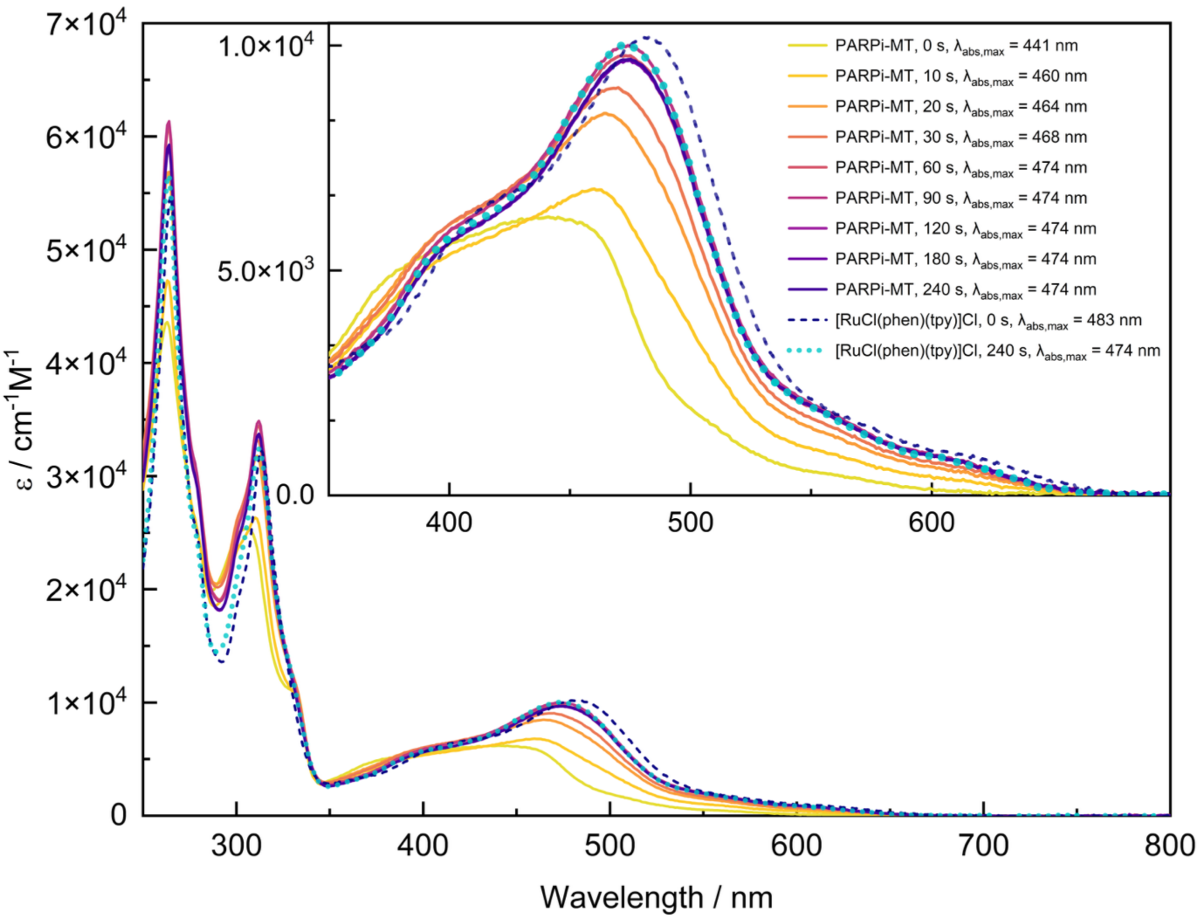

Figure S22. Qualitative assessment of the photocleavage efficiency of **PARPi-MT**. A solution of **PARPi-MT** in PBS containing 5% MeOH (10 µM) was irradiated at 420 nm using a Lumidox® II LED controller equipped with a 96-position single wavelength LED array (2.9 W total radiant power, 93.75 mW/cm^2). UV-Vis absorption spectra were recorded at defined total irradiation times (t = 0, 10, 20, 30, 60, 90, 120, 180 and 240 s) to monitor spectral changes associated with photocleavage. Photoconversion was indicated by a red shift in the absorption maximum, consistent with the formation of the aqua complex $[Ru(H_2O)(phen)(tpy)]^{2+}$. To validate this, a solution of [RuCl(phen)(tpy)]Cl in PBS containing 1% MeOH (10 µM) was measured before and after 240 s of irradiation under identical conditions. Complete conversion of **PARPi-MT** to the aqua complex was observed after 60 s, as evidenced by the appearance of an absorption maximum at 474 nm, matching that of the irradiated [RuCl(phen)(tpy)]Cl solution.

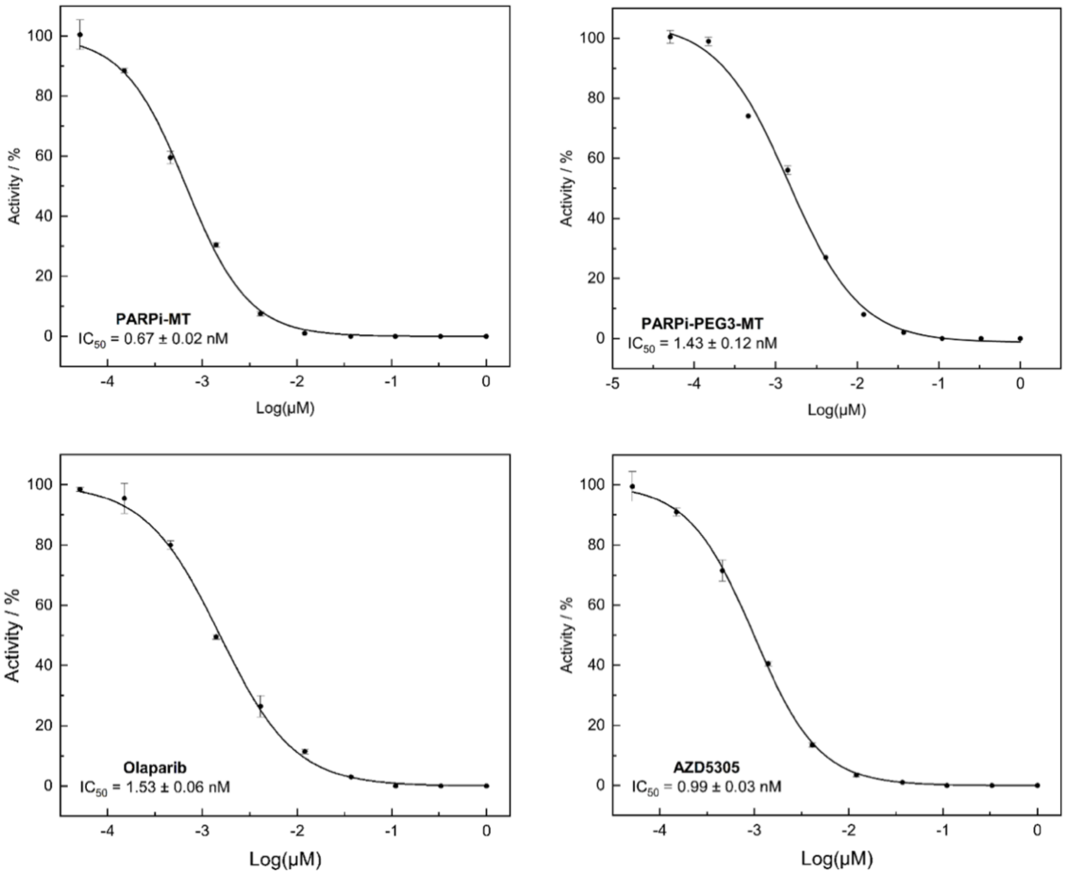

Figure S23. Dose-response curves for determining the PARP1 IC_{50} values of **PARPi-MT**, **PARPi-PEG3-MT**, Olaparib and AZD5305. Data produced by BPS Bioscience, San Diego, CA, through a colorimetric Strep-HRP assay with a histone substrate and biotin-labeled NAD^+ at ten different concentrations ranging from 0.05 nM to 1 μM.

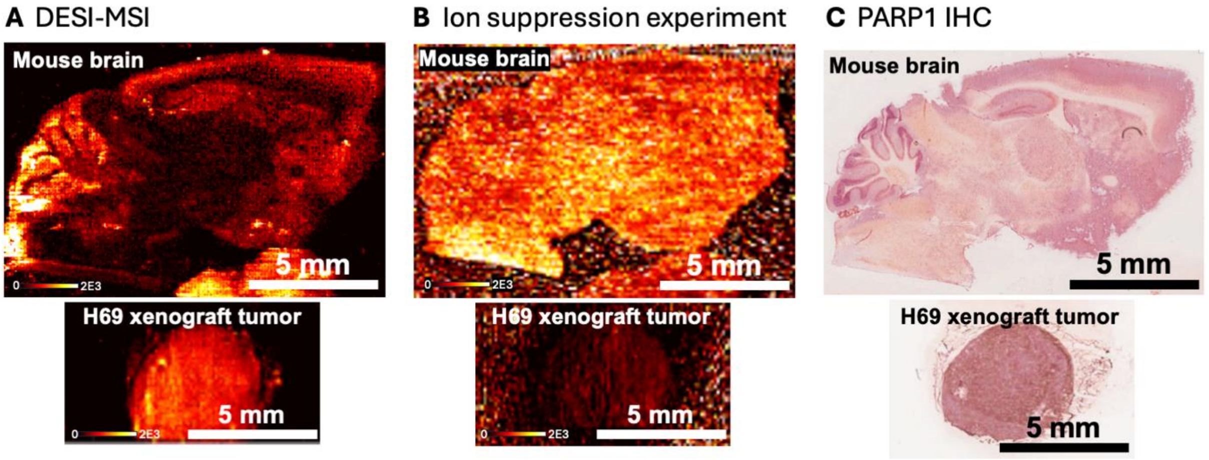

Figure S24. Analysis of ion suppression of $[Ru(CF_3COO)(phen)(tpy)]^+$ in distinct tissue regions of mouse brain and H69 xenograft tumor via DESI-MSI. A: **PARPi-MT**-stained tissue section analyzed for $[Ru(CF_3COO)(phen)(tpy)]^+$ distribution (*m/z* 628.0522 ± 3 ppm; TIC-normalized; spatial resolution; X,Y = 60 μm) via DESI-MSI. B: Control tissue section sprayed with an aqueous solution of 10 μM [RuCl(phen)(tpy)]Cl and 10 μM TFA using a HTX TM-sprayer™ and analyzed for ion suppression of $[Ru(CF_3COO)(phen)(tpy)]^+$ signal (*m/z* 628.0538 ± 3 ppm; TIC-normalized; spatial resolution; X,Y = 65 μm) in DESI-MSI. C: Corresponding PARP1 IHC of the same tissue samples for comparison.

Table S1. List of metabolites increased in mouse brain tissue section post **PARPi-MT** staining, tentatively identified based on exact mass.

#	Detected *m/z*	Name	Formula	Monoisotopic mass	Adduct	Adduct *m/z*	Δ_{ppm}
**1**	203.2225	Spermine	$C_{10}H_{26}N_4$	202.2157	M+H	203.2230	3
**2**	282.2782	Oleamide	$C_{18}H_{35}NO$	281.2719	M+H	282.2791	3
**3**	283.2623	FA(18:1)	$C_{18}H_{34}O_2$	282.2559	M+H	283.2632	3
	305.2441				M+Na	305.2451	3
**4**	300.2888	Palmitoylethanolamide	$C_{18}H_{37}NO_2$	299.2824	M+H	300.2897	3
**5**	303.2299	FA(18:2)	$C_{18}H_{32}O_2$	280.2402	M+Na	303.2294	1
**6**	316.2837	Dehydrophytosphingosine	$C_{18}H_{37}NO_3$	315.2773	M+H	316.2846	3
**7**	321.2390	FA(18:1;O)	$C_{18}H_{34}O_3$	298.2508	M+Na	321.2400	3
**8**	323.2547	FA(18:0;O)	$C_{18}H_{36}O_3$	300.2664	M+Na	323.2557	3
**9**	337.2702	FA(19:0;O)	$C_{19}H_{38}O_3$	314.2821	M+Na	337.2713	3
**10**	357.2988	MG(18:1)	$C_{21}H_{40}O_4$	356.2927	M+H	357.2999	3
**11**	385.3300	MG(20:1)	$C_{23}H_{44}O_4$	384.3240	M+H	385.3312	3
**12**	395.2755	DG(18:0)	$C_{21}H_{40}O_5$	372.2876	M+Na	395.2768	3
**13**	400.3409	Palmitoylcarnitine	$C_{23}H_{45}NO_4$	399.3349	M+H	400.3421	3
**14**	407.3258	*N*-Palmitoyl lysine	$C_{22}H_{44}N_2O_3$	384.3352	M+Na	407.3244	3
**15**	411.2704	MG(18:1;O2)	$C_{21}H_{40}O_6$	388.2825	M+Na	411.2717	3
**16**	419.3846	FA(26:0)	$C_{26}H_{52}O_2$	396.3967	M+Na	419.3859	3
**17**	502.2892	LysoPE(18:1)	$C_{23}H_{46}NO_7P$	479.3012	M+Na	502.2904	2
**18**	530.3212	LysoPE(20:1)	$C_{25}H_{50}NO_7P$	507.3325	M+Na	530.3217	1
**19**	544.3358	LysoPC(18:1)	$C_{26}H_{52}NO_7P$	521.3481	M+Na	544.3374	3
**20**	548.2730	LysoPE(22:6)	$C_{27}H_{44}NO_7P$	525.2855	M+Na	548.2748	3
**21**	597.5613	CE(14:0)	$C_{41}H_{72}O_2$	596.5532	M+H	597.5605	1
**22**	808.5742	DG(LTE4/20:0)	$C_{46}H_{81}NO_8S$	807.5683	M+H	808.5756	2
**23**	809.5765	SM(d18:0/20:3(8Z,11Z,14Z)-2OH(5,6))	$C_{43}H_{83}N_2O_8P$	786.5887	M+Na	809.5779	2

References

1. M. Loos, C. Gerber, F. Corona, J. Hollender and H. Singer, Accelerated Isotope Fine Structure Calculation Using Pruned Transition Trees, *Analytical Chemistry* **2015**, 87, 5738-5744.
2. H. Rauch, C. Kitzberger, K. Janghu, P. Hawarihewa, N. T. Nguyen, Y. Min, S. Ballke, K. Steiger, W. A. Weber and S. Kossatz, Combining [177Lu]Lu-DOTA-TOC PRRT with PARP inhibitors to enhance treatment efficacy in small cell lung cancer, *European Journal of Nuclear Medicine and Molecular Imaging* **2024**, 51, 4099-4110.
3. R. Adusumilli and P. Mallick, in *Proteomics: Methods and Protocols*, eds. L. Comai, J. E. Katz and P. Mallick, Springer New York, New York, NY, 2017, DOI: 10.1007/978-1-4939-6747-6_23, pp. 339-368.
4. A. M. Race, I. B. Styles and J. Bunch, Inclusive sharing of mass spectrometry imaging data requires a converter for all, *Journal of Proteomics* **2012**, 75, 5111-5112.
5. G. Robichaud, K. P. Garrard, J. A. Barry and D. C. Muddiman, MSiReader: An Open-Source Interface to View and Analyze High Resolving Power MS Imaging Files on Matlab Platform, *Journal of The American Society for Mass Spectrometry* **2013**, 24, 718-721.
6. S. Tortorella, P. Tiberi, A. P. Bowman, B. S. R. Claes, K. Ščupáková, R. M. A. Heeren, S. R. Ellis and G. Cruciani, LipostarMSI: Comprehensive, Vendor-Neutral Software for Visualization, Data Analysis, and Automated Molecular Identification in Mass Spectrometry Imaging, *Journal of the American Society for Mass Spectrometry* **2020**, 31, 155-163.
7. W. Dong, J. Tang, L. Zhao, F. Chen, L. Deng and M. Xian, The visible-light-driven transfer hydrogenation of nicotinamide cofactors with a robust ruthenium complex photocatalyst, *Green Chemistry* **2020**, 22, 2279-2287.
8. K. Kitamura, H. Itoh, K. Sakurai, S. Dan and M. Inoue, Target Identification of Yaku'amide B and Its Two Distinct Activities against Mitochondrial FoF1-ATP Synthase, *Journal of the American Chemical Society* **2018**, 140, 12189-12199.
9. M. P. Jacinto, S. D. Fried and M. M. Greenberg, Intracellular Formation of a DNA Damage-Induced, Histone Post-Translational Modification Following Bleomycin Treatment, *Journal of the American Chemical Society* **2022**, 144, 7600-7605.
