# Supplementary material for: A Small Molecule Drug-Based Ru(II) Polypyridine Mass-Tag for Multimodal Imaging of Tissue Samples
Source: ACS Cent Sci. 2025 Oct 21;11(11):2230–9. doi: 10.1021/acscentsci.5c01381 (PMC12670285; doi:10.1021/acscentsci.5c01381)
Supplement: Supplementary file 2 [file oc5c01381_si_002.pdf]

oc-2025-01381v.R1

Name: Peer Review Information for "A small molecule drug-based Ru(II) polypyridine mass-tag for multimodal imaging of tissue samples"

First Round of Reviewer Comments

Reviewer: 1

Comments to the Author

Casini, Strittmatter, and colleagues have reported a new ruthenium(II) complex conjugated with a small molecule drug, aimed at enabling targeted bimodal imaging of PARP1 in xenograft tumors and mouse brain tissues. This complex has been successfully employed for both mass spectrometry and fluorescence imaging, demonstrating greater potency than the parent drug compound. These findings suggest that the conjugation of established drugs with metal-based fragments represents a promising strategy for developing new bimodal imaging probes. This work builds on a previously published study (Chem. Commun. 2020, 56, 5941), and the insights gained are valuable for advancing mass spectrometry imaging and the design of new small-molecule-based metal complexes.

I believe this manuscript will be of significant interest to the readers of ACS Central Science, and I recommend it for publication with a few minor suggestions:

1. Did the authors assess the stability of the probe in the presence of other thiol-based compounds? Specifically, could the N-acetyl-L-cysteine wash be substituted with the thioether ligand of the ruthenium(II) complex?

2. While the authors demonstrate that PARP1 labeling can be achieved with a ruthenium(II) conjugated Olaparib, it would be interesting to explore whether other disease biomarkers could be visualized using this strategy with different small molecule-based drugs. A discussion on this potential would be beneficial.

3. Additional information regarding the photocleavage efficiency, even if qualitative, would enhance the manuscript.

4. The ruthenium(II) terpyridine unit is known for its weak emission intensities. To improve sensitivity in confocal imaging, the authors might consider the [Ru(N^N)2L2] configuration. Could the authors provide insights on this possibility and suggest alternative designs for future investigations?

5. In hindsight, the steric bulkiness of the ruthenium moiety may be a disadvantage. While the PEG spacer arm can possibly alleviate potential steric hindrance, it appears that this modification does not enhance the affinity of the complex to PARP1 as expected. Further explanations are needed to clarify this observation.

Reviewer: 2

Comments to the Author

In this manuscript, authors introduced a class of multimodal, photocleavable, metal-based MTs derived from a small-molecule drug inhibitor. PARPi-MT, a Ru(II)-based MT incorporating the PARP1 inhibitor Olaparib as binding motif, enables spatial visualization of PARP1 in murine brain and xenograft tumor tissues using both DESI-MSI and fluorescence microscopy. By employing small-molecule drugs as binding motifs and leveraging the structural flexibility of the Ru(II) reporter moiety, this approach offers an alternative to Abs- and peptide-based systems, with strong potential for high multiplexing capabilities. But the following issues should be addressed before publication.

1. Compared to PARPi-MT, the PARPi-PEG3-MT exhibited lower affinity for PARP1. Could the authors explain the reasons for this from a structural perspective?

2. When using PARPi-MT for PARP1 imaging in tissue sections, in addition to selecting model tissues expressing PARP1, should the authors also demonstrate a significantly reduced imaging signal in the PARP1 knockout systems as proof of specificity?

3. When using PARPi-MT for dual-modal imaging (MSI and fluorescence, Figure 4), the results of the colocalization analysis between the two imaging modalities, such as the Pearson correlation coefficient, should be provided to quantitatively demonstrate their spatial concordance.

4. Why did the authors choose Ru(II) polypyridine complexes as the core of the mass tag? What specific advantages does it offer in terms of ionization efficiency, background noise, and photostability in MALDI-MS imaging compared to other metal complexes (e.g., Ir(III) or Os(II))?

Author's Response to Peer Review Comments:

Dear Editor,

Please find herewith uploaded the revised version of our manuscript in which we have addressed the reviewers' comments as detailed in the "Answers to Reviewers" file.

We hope that the paper is now suitable for acceptance in ACS Central Science.

Looking forward to hearing from you.

best regards,

Angela Casini

**Answers to Reviewers:**

# Reviewer: 1

*Recommendation: Publish in ACS Central Science after minor revisions noted.*

*Comments:*
*Casini, Strittmatter, and colleagues have reported a new ruthenium(II) complex conjugated with a small molecule drug, aimed at enabling targeted bimodal imaging of PARP1 in xenograft tumors and mouse brain tissues. This complex has been*

*successfully employed for both mass spectrometry and fluorescence imaging, demonstrating greater potency than the parent drug compound. These findings suggest that the conjugation of established drugs with metal-based fragments represents a promising strategy for developing new bimodal imaging probes. This work builds on a previously published study (Chem. Commun. 2020, 56, 5941), and the insights gained are valuable for advancing mass spectrometry imaging and the design of new small-molecule-based metal complexes.*

*I believe this manuscript will be of significant interest to the readers of ACS Central Science, and I recommend it for publication with a few minor suggestions:*

*1. Did the authors assess the stability of the probe in the presence of other thiol-based compounds? Specifically, could the N-acetyl-L-cysteine wash be substituted with the thioether ligand of the ruthenium(II) complex?*

**Answer:** To address this comment, we have added an NMR study of the reactivity of the **PARPi-MT** with N-acetyl-L-cysteine in the revised manuscript. As shown in Figure S16 the mass-tag is stable over 24 h incubation.

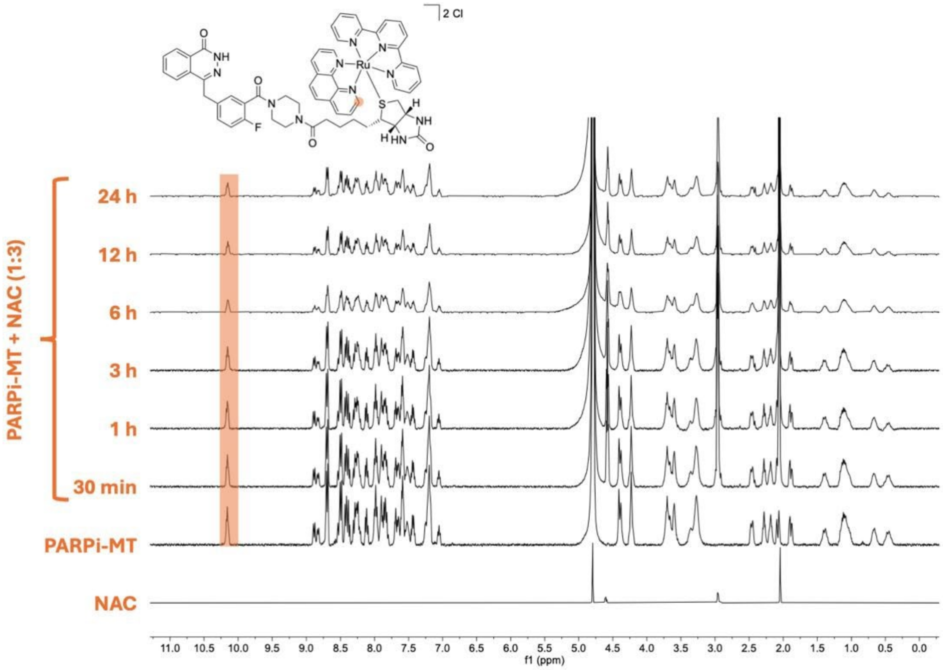


**Figure S16.** The stability of **PARPi-MT** in $D_2O$ in the presence of NAC was assessed. To a solution of **PARPi-MT** in $D_2O$ (3.5 mM, 300 µL), 10 µL of a NAC solution (300 mM) in $D_2O$ was added, resulting in a final mixture with **PARPi-MT** and NAC at a 1:3 molar ratio. The signal corresponding to the marked proton on the 1,10-phenanthroline ligand was monitored at defined time intervals (t = 30 min, 1, 3, 6, 12, 24 h) over a period of 24 h. No change in peak integral or spectral profile was observed, indicating that **PARPi-MT** remains stable in the presence of NAC under ambient conditions.

*2. While the authors demonstrate that PARP1 labeling can be achieved with a ruthenium(II) conjugated Olaparib, it would be interesting to explore whether other disease biomarkers could be visualized using this strategy with different small molecule-based drugs. A discussion on this potential would be beneficial.*

**Answer:** We fully agree with this comment, and we have added a section on other possible small-molecule-based Ru(II) mass-tags (MTs). These could include, for example, inhibitors of disease-relevant enzymes. Ongoing studies in our group are devoted to the development of FAP-targeted MTs. In fact, the serine protease fibroblast activation protein (FAP) allows a selective targeting of a variety of tumors, and recently, highly selective small-molecule inhibitors (FAPi) have been successfully incorporated in the design of radiopharmaceuticals for theranostic applications. This information and related references have been added to the conclusion section of the revised manuscript.

*3. Additional information regarding the photocleavage efficiency, even if qualitative, would enhance the manuscript.*

**Answer:** The photocleavage efficiency of **PARPi-MT** was also qualitatively assessed by irradiating a solution of the MT at 420 nm and monitoring the spectral changes over time (Figure S22). UV-Vis absorption spectra were recorded at defined total irradiation times (t = 0, 10, 20, 30, 60, 90, 120, 180 and 240 s) to track the progression of photocleavage. Photoconversion was indicated by a red shift in the absorption maximum, consistent with the formation of the aqua complex $[Ru(H_2O)(phen)(tpy)]^{2+}$, as previously reported in the literature. To validate this, a 10 μM solution of [RuCl(phen)(tpy)]Cl in PBS containing 1% MeOH was measured before and after 240 s of irradiation under identical conditions. Complete conversion of **PARPi-MT** to the aqua complex was observed after 60 s, as evidenced by the appearance of an absorption maximum at 474 nm, matching that of the irradiated [RuCl(phen)(tpy)]Cl solution.

Discussion is included in the revised manuscript.

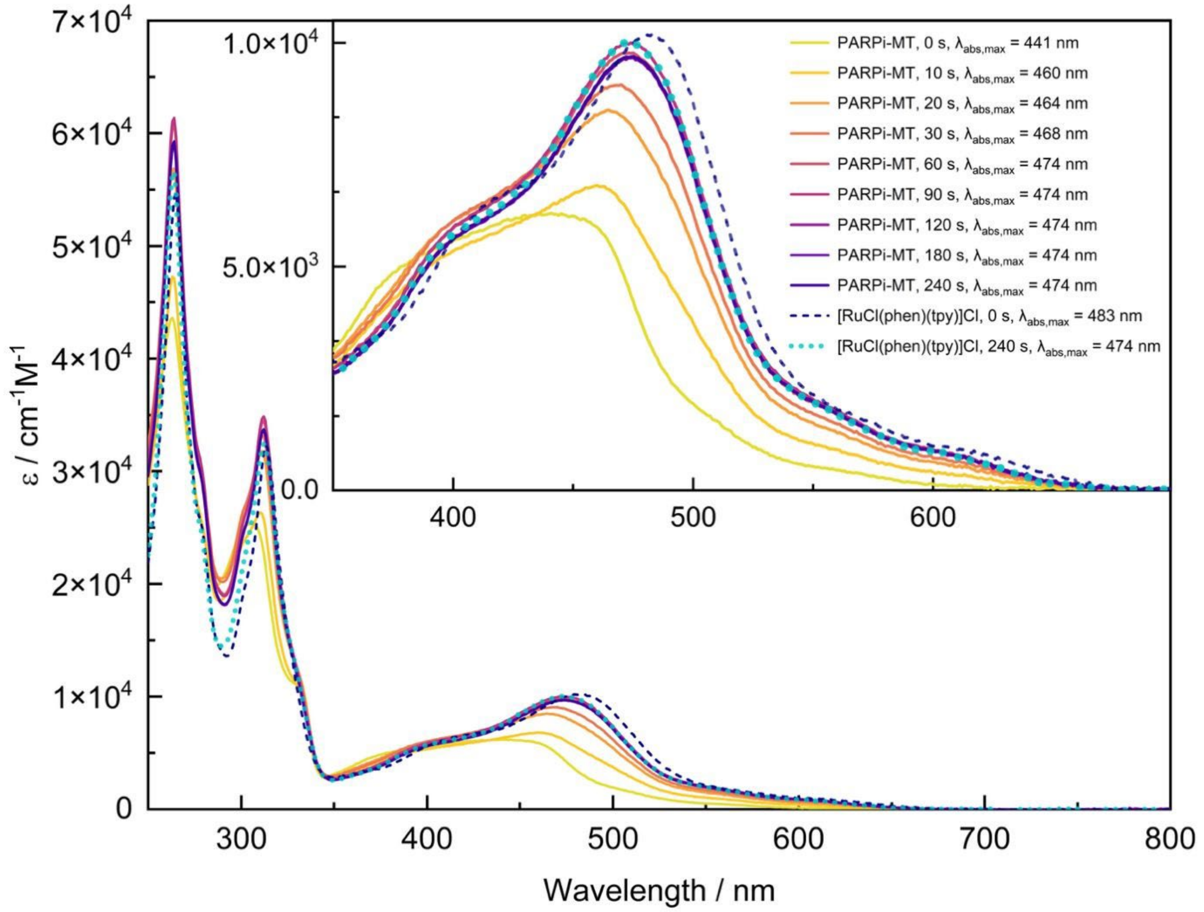


**Figure S1.** Qualitative assessment of the photocleavage efficiency of **PARPi-MT**.

*4. The ruthenium(II) terpyridine unit is known for its weak emission intensities. To improve sensitivity in confocal imaging, the authors might consider the [Ru(N^N)2L2] configuration. Could the authors provide insights on this possibility and suggest alternative designs for future investigations?*

**Answer:** We thank the reviewer for this comment, since it allows us to better clarify the selection of the Ru(II) moiety in our MT approach. Upon photoexcitation, certain ruthenium polypyridyl compounds are known to selectively photosubstitute one ligand of the coordination sphere by a solvent molecule. We postulated that the 'uncaging' of the Ru(II) MT upon UVlight activation could facilitate its detection, providing a *fingerprint signal* in the MS spectrum. This was demonstrated in our previous work (Han et al. *Chem. Commun.* 2020). Moreover, as explained in our introduction, unlike organic reporter groups, which can be challenging to differentiate from endogenous molecules, ruthenium offers a unique isotopic pattern distribution that facilitates unambiguous identification in complex MSI datasets. The Ru(II) moiety is positively charged, easily ionizable and amenable to ligand modification, enabling potential multiplexing capabilities.

In the present manuscript, in addition to the aforementioned capabilities, we decided to also explore the luminescence properties of the Ru(II) moiety for fluorescence imaging.

Coming back to the reviewer's comment, the Ru(II) polypyridyl compound's propensity for photoactivation *versus* fluorescence is determined by its molecular structure and electronic properties, which dictate the dominant pathway of excited-state deactivation.

The classical mechanism of photosubstitution in polypyridyl ruthenium(II) compounds starts from $^3$MLCT (metal-to-ligand charge-transfer triplet) excited states. While these are typically responsible for the phosphorescence, electron-transfer, or energy-transfer processes observed with photoinert compounds such as $[Ru(bpy)_3]^{2+}$, they can also be thermally promoted to a metal-centered ($^3$MC) triplet excited states that lies close enough in energy. Population of the $^3$MC causes the ejection of a ligand.

In contrast, Ru(II) polypyridyl complexes with high photoluminescence quantum yields efficiently emit light from their triplet excited states, providing long-lived and bright signals for bioimaging. Overall, in many instances, the two features (photoactivation *versus* fluorescence) do not converge in the same molecule and design concept.

Presently, we are trying to optimize the Ru(II) MT for photosubstitution and multiplexing; therefore, we will focus on the family of [RuL(tpy)(N^N)], where tpy = 2,2′:6′,2″-terpyridine, N^N is a variety of bidentate ligands, and L is the "caged" monodentate pyridyl ligand that can be released upon irradiation. In parallel, we will evaluate the effects of ligand substitution on the fluorescence quantum yield. We have added some of these considerations in the revised version of the manuscript (Conclusions section).

Finally, the suggested use of [Ru(N^N)2L2] complexes featuring two monodentate ligands may result in the uncaging of two different parts of the reporter group, which would increase speciation in the MSI process and only add complexity to the spectra interpretation.

*5. In hindsight, the steric bulkiness of the ruthenium moiety may be a disadvantage. While the PEG spacer arm can possibly alleviate potential steric hindrance, it appears that this modification does not enhance the affinity of the complex to PARP1 as expected. Further explanations are needed to clarify this observation.*

**Answer:** Concerning the PEG-bearing mass-tag, it should be noted that the PARP-1 inhibitory potency of this compound is still extremely high and comparable to the one of Olaparib ($IC_{50}$ = 1.43 ± 0.12 nM vs 1.53 ± 0.06 nM, respectively). Therefore, the presence of the longer PEG 'arm' is still well tolerated by the protein active site. Of course, when we had

to choose the mass-tag to pursue the first experiments, we opted for the most potent inhibitor; whose lower IC50 value (0.67 ± 0.02 nM) may be due to other favorable interactions of the cationic Ru(II) moiety with the protein surface. While we will keep a close eye on structure-activity relationships in future work, it is beyond the scope of this manuscript to unravel these subtle relationships in detail.

# Reviewer: 2

*Recommendation: Publish in ACS Central Science after minor revisions noted.*

*Comments:*
*In this manuscript, authors introduced a class of multimodal, photocleavable, metal-based MTs derived from a small-molecule drug inhibitor. PARPi-MT, a Ru(II)-based MT incorporating the PARP1 inhibitor Olaparib as binding motif, enables spatial visualization of PARP1 in murine brain and xenograft tumor tissues using both DESI-MSI and fluorescence microscopy. By employing small-molecule drugs as binding motifs and leveraging the structural flexibility of the Ru(II) reporter moiety, this approach offers an alternative to Abs- and peptide-based systems, with strong potential for high multiplexing capabilities. But the following issues should be addressed before publication.*

*1. Compared to PARPi-MT, the PARPi-PEG3-MT exhibited lower affinity for PARP1. Could the authors explain the reasons for this from a structural perspective?*

**Answer:** As mentioned in the answer to reviewer 1, the PEG-bearing mass-tag features comparable PARP-1 inhibitory potency to Olaparib ($IC_{50}$ = 1.43 ± 0.12 nM vs 1.53 ± 0.06 nM, respectively). Therefore, the presence of the longer PEG 'arm' is still well tolerated by the protein active site. For the purpose of our work, we could have used both Ru(II) mass-tags, but we decided to go on with the one that had an even better affinity than Olaparib for the presented MSI studies.

*2. When using PARPi-MT for PARP1 imaging in tissue sections, in addition to selecting model tissues expressing PARP1, should the authors also demonstrate a significantly reduced imaging signal in the PARP1 knockout systems as proof of specificity?*

**Answer:** As PARP1 is an essential nuclear enzyme and it is expressed in all human tissues. Unfortunately, we do not have access to KO negative-control tissue for testing. In our experiments, we therefore, selected samples with heterogeneous PARP1 expression across tissues to demonstrate specificity. In addition, as shown in Figure S24, we conducted a control experiment in which tissue samples were coated with a uniform, fine layer of $[Ru(CF_3COO)(phen)(tpy)]^+$. This confirmed that the observed signal differences are not due

to regional ion-suppression effects. Together, these measures compensate for the absence of a conventional negative control, which is not feasible given the nature of our target.

*3. When using PARPi-MT for dual-modal imaging (MSI and fluorescence, Figure 4), the results of the colocalization analysis between the two imaging modalities, such as the Pearson correlation coefficient, should be provided to quantitatively demonstrate their spatial concordance.*

**Answer:** Both MSI and confocal spectroscopy are non-quantitative and feature different resolutions, different background noise levels and different data formats. Therefore, direct quantitative comparison and a meaningful correlation coefficient are computationally not straightforward to obtain. In a modified version of Figure 4, we have now assed an overlaid image of both the MSI and fluorescence images to illustrate the colocalization of the two imaging modalities to the same tissue features.

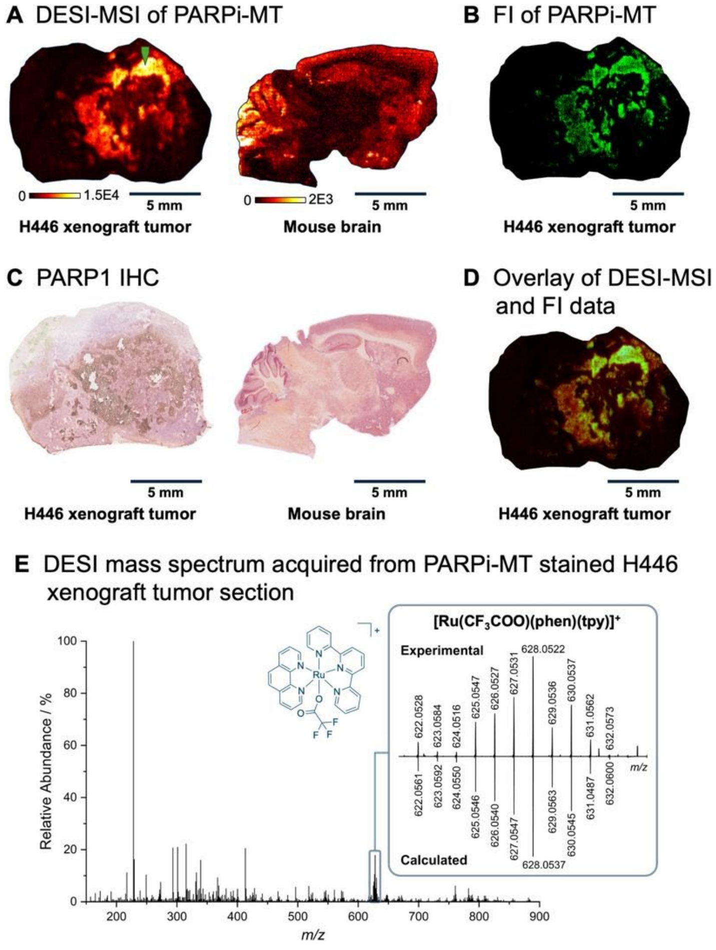


**Figure 4.** A: DESI-MSI of a **PARPi-MT** stained H446 xenograft tumor and mouse brain section, depicting the spatial distribution of $[Ru(CF_3COO)(phen)(tpy)]^+$

(*m/z* 628.0521 ± 3 ppm; total ion current (TIC)-normalized; spatial resolution: X,Y = 60 µm). B: Confocal fluorescence microscopy image of the same H446 xenograft tumor section used for DESI-MSI, employing a 600-725 nm channel mode with false-color representation (green). C: IHC of corresponding adjacent tissue sections, stained for PARP1. D: Overlay of DESI-MSI (in red) and FI images (in green). E: A DESI mass spectrum recorded at the marked spot (green arrow) of the H446 xenograft tumor shows the isotope pattern distribution of $[Ru(CF_3COO)(phen)(tpy)]^+$ in comparison to the calculated isotopic pattern.

*4. Why did the authors choose Ru(II) polypyridine complexes as the core of the mass tag? What specific advantages does it offer in terms of ionization efficiency, background noise, and photostability in MALDI-MS imaging compared to other metal complexes (e.g., Ir(III) or Os(II))?*

**Answer:** As already discussed above, the main design concept of our mass-tag includes the 'uncaging' of the metal-based MT upon UV-light activation, which is pivotal to facilitate its detection, providing a *fingerprint signal* in the MS spectrum. From the literature, one of the most used systems to achieve photo-substitution reactions are Ru(II) polypyridyl complexes. This reactivity is not present (or extremely rare) in other metal complexes. For example, photosubstitution on Os(II) complexes is very rare and very slow. We have included these considerations and references in the introduction of the revised manuscript.
